# Supplementary figures and images for: SAMD1 suppresses epithelial–mesenchymal transition pathways in pancreatic ductal adenocarcinoma
Source: PLoS Biol. 2024 Aug 13;22(8):e3002739. doi: 10.1371/journal.pbio.3002739 (PMC11343471; doi:10.1371/journal.pbio.3002739)

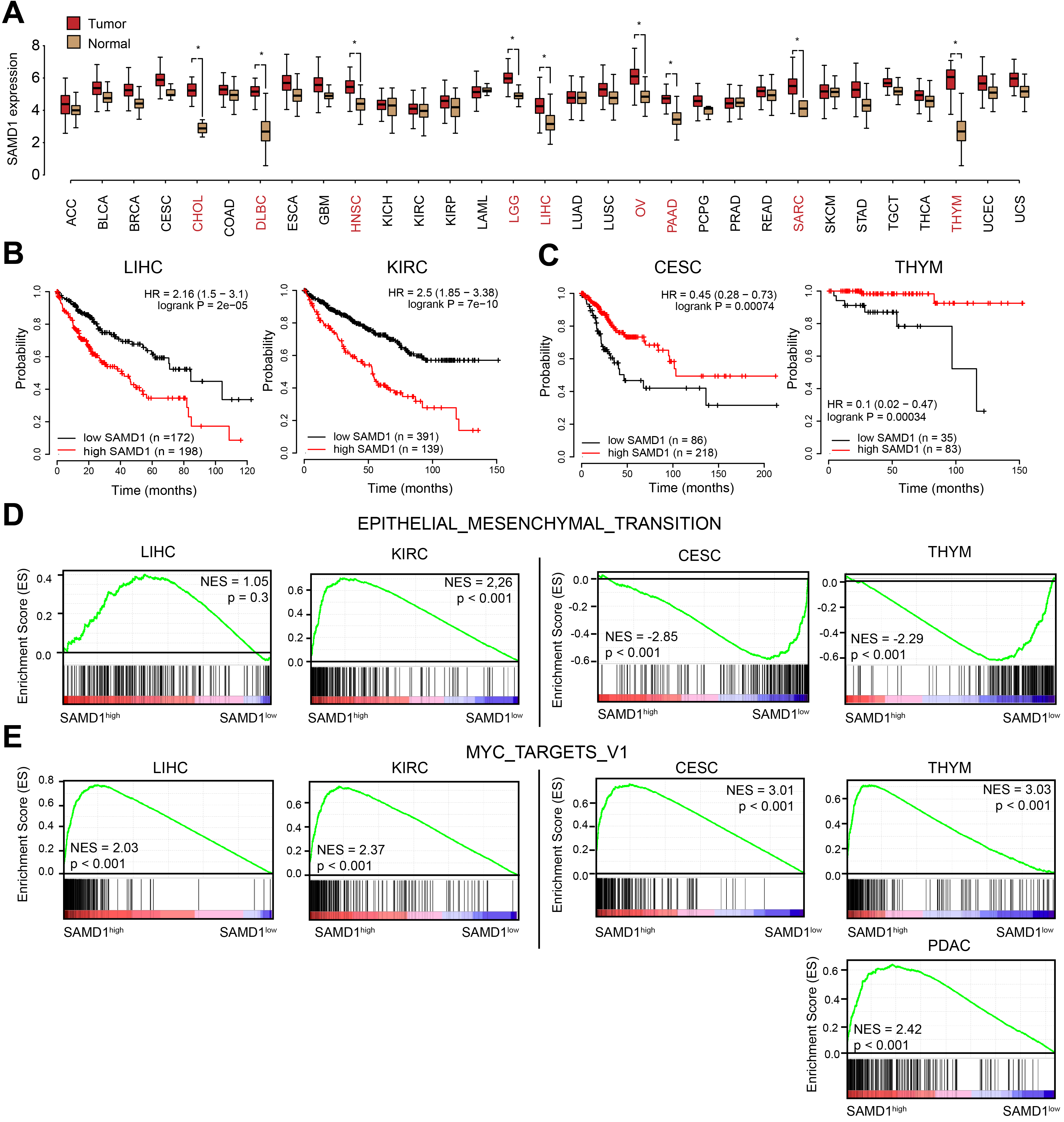

Supplement: S1 Fig — (A) Expression of SAMD1 in cancer versus normal tissues. Data from TCGA [20] and visualized via GePIA [21]. Cancer types highlighted in red indicate significantly up-regulated SAMD1 expression. (B) Kaplan—Meier survival curves (overall survival) in liver hepatocellular carcinoma (LIHC) and kidney renal clear cell carcinoma (KIRC), using auto-selected cut-offs. High SAMD1 expression correlates with a worse prognosis. (C) Kaplan—Meier survival curves (overall survival) in cervical cancer (CESC) and thymoma (THYM). High SAMD1 expression correlates with a better prognosis. Data in (B) and (C) are derived from TCGA and visualized via the Kaplan—Meier plotter tool [22] using auto-selected cut-off. (D) GSEA of the epithelial—Mesenchymal transition (EMT) pathway in the cancer types presented in (B) and (C). (E) GSEA of the MYC target genes in the cancer types presented in (B) and (C) and of PDAC. In (D) and (E), tissue samples with high SAMD1 expression are compared to samples with low SAMD1 expression. The data underlying this figure is available in S1 Data. (TIF) [file pbio.3002739.s001.tif]

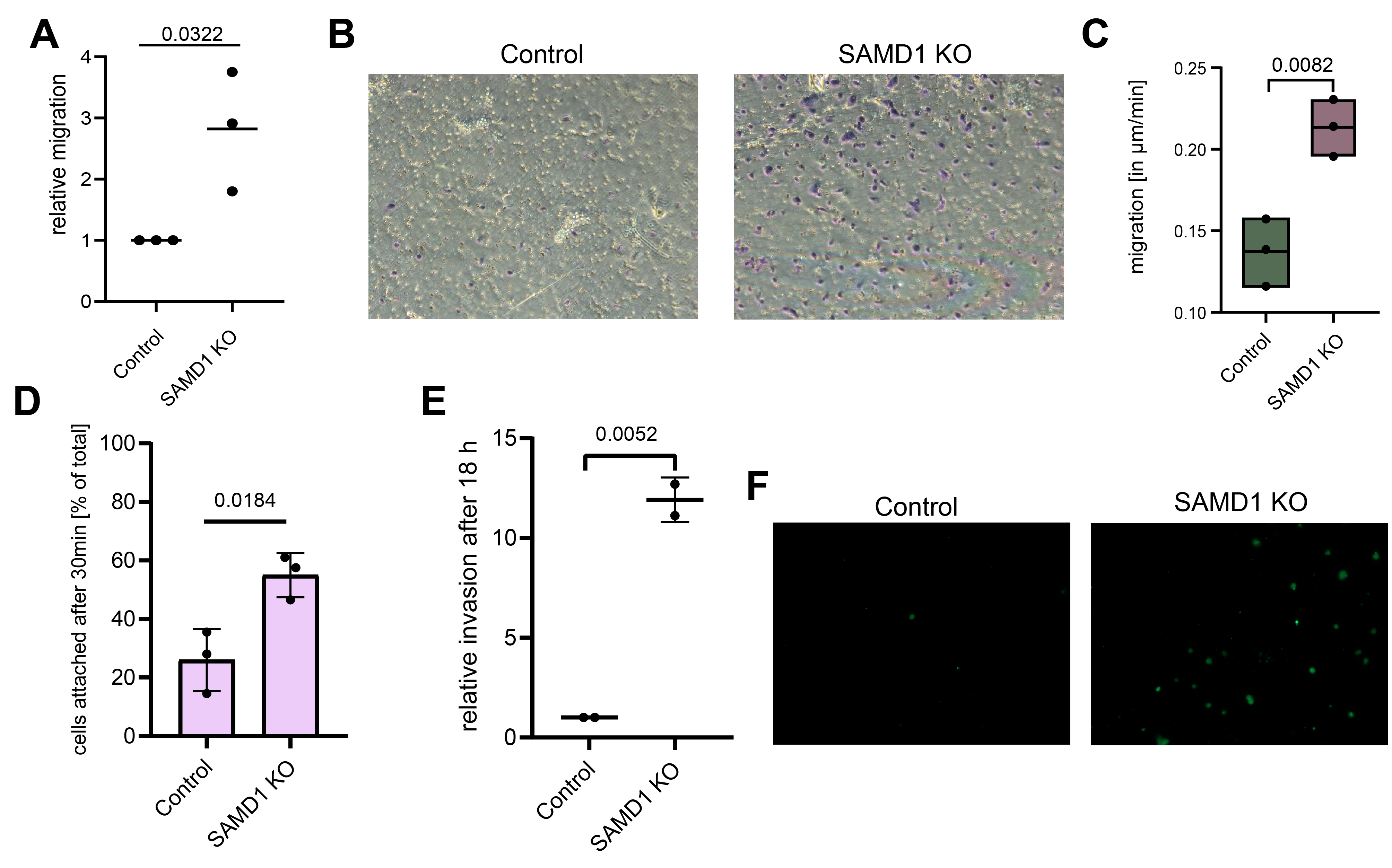

Supplement: S2 Fig — (A) Transwell migration assay of PaTu8988t control and SAMD1 KO cells. Data represent the mean ± SD of 3 biological replicates. Significance was analyzed using Student’s t test. (B) Representative crystal violet staining of 1 transwell migration assay. (C) Migration of PaTu8988t control and SAMD1 KO cells in μm/min based on time-lapse analysis. See also S1 and S2 Movies. Data represent the mean ± SD of 3 biological replicates. Significance was analyzed using Student’s t test. (D) Adhesion assay of PaTu8988t control and SAMD1 KO cells. Data represent the mean ± SD of 3 biological replicates. Significance was analyzed using Student’s t test. (E) Invasion assay of PaTu8988t control and SAMD1 KO cells. Data represent the mean ± SD of 2 biological replicates. Significance was analyzed using Student’s t test. (F) Representative cell tracker staining of 1 adhesion assay. The data underlying this figure is available in S1 Data. (TIF) [file pbio.3002739.s002.tif]

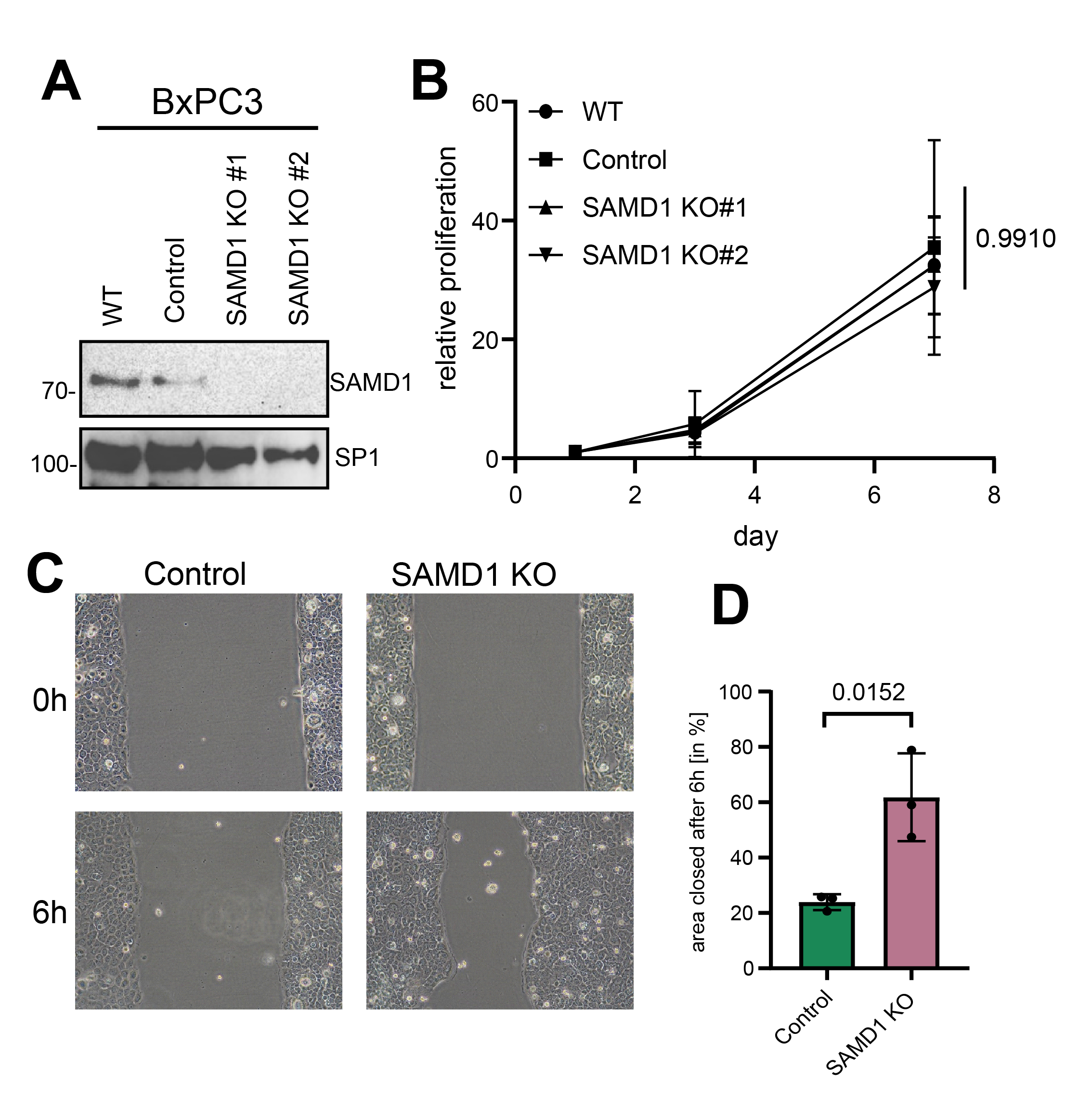

Supplement: S3 Fig — (A) Western blot showing BxPC3 wild-type cells, control cells, and 2 different SAMD1 knockout clones. (B) Proliferation assay of BxPC3 wild-type cells, control cells, and 2 different SAMD1 knockout clones. Data represent the mean ± SD of 3 biological replicates. Significance was analyzed using one-way ANOVA. (C) Representative picture of one wound healing assay of BxPC3 control cells and one SAMD1 knockout clone. (D) Quantification of the wound healing assay from (C). Data represent the mean ± SD of 3 biological replicates, and significance was analyzed using Student’s t test. The data underlying this figure is available in S1 Data. (TIF) [file pbio.3002739.s003.tif]

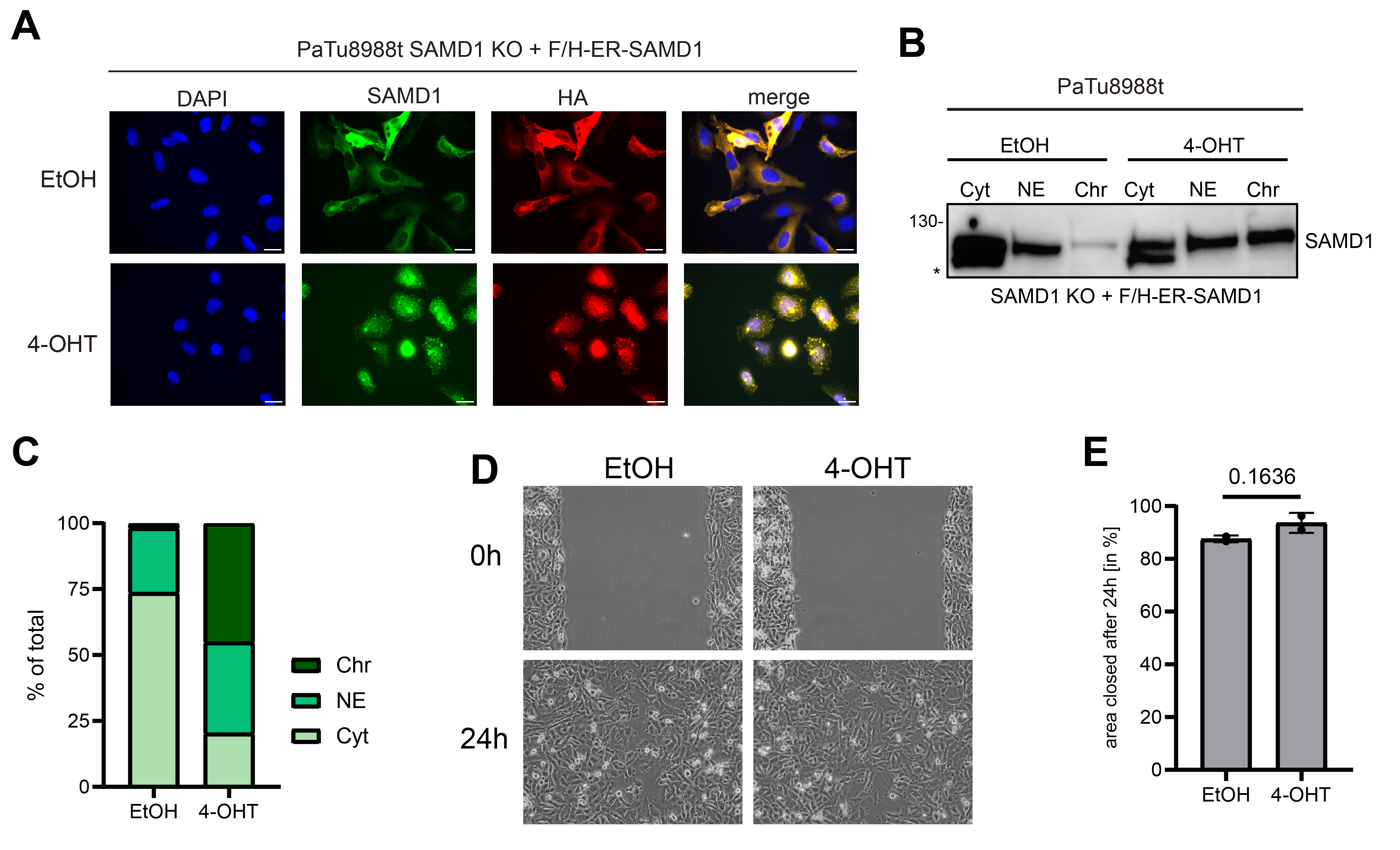

Supplement: S4 Fig — (A) Immunofluorescence of PaTu8988t SAMD1 knockout cells with or without induction of SAMD1 rescue, Bar = 20 μm. (B) Western blot after fractionation of PaTu8988t SAMD1 knockout cells with or without induction of SAMD1 rescue. (C) Quantification of (B). (D) Representative picture of one wound healing assay of PaTu8988t SAMD1 KO cells expressing FH-ER with and without 4-OHT induction. (E) Quantification of the wound healing assay from (D). Data represent the mean ± SD of 2 biological replicates, and significance was analyzed using Student’s t test. The data underlying this figure is available in S1 Data. (TIF) [file pbio.3002739.s004.tif]

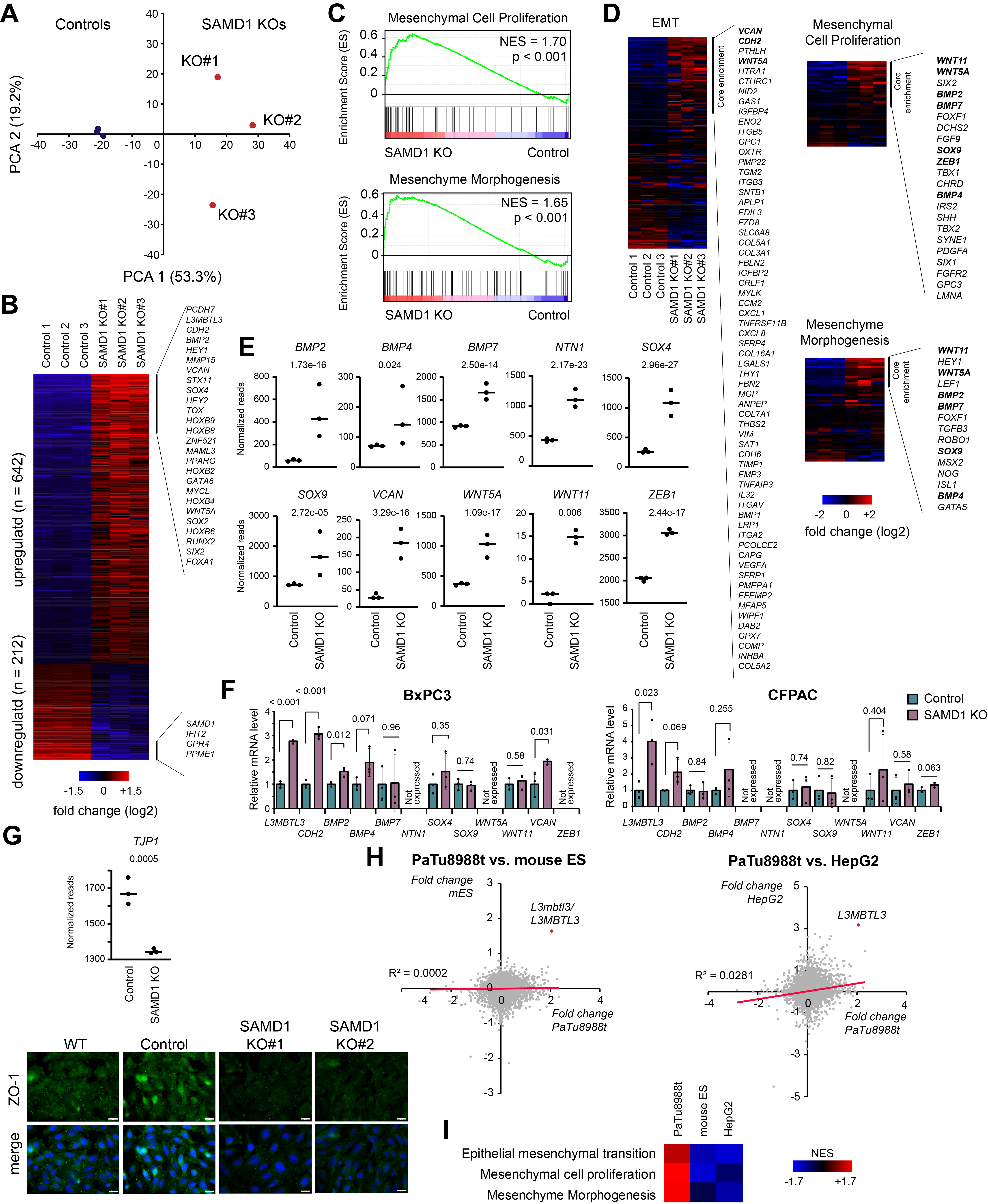

Supplement: S5 Fig — (A) Principal component analysis (PCA) of RNA-Seq data upon SAMD1 KO. Three clonally independent SAMD1 KO clones were used. (B) Heatmap of the significantly dysregulated genes. Examples of the most dysregulated genes are shown on the right. (C) GSEA analysis of mesenchymal-related pathways in SAMD1 KO versus control cells (D). Heatmap of genes in genesets from GSEA analysis in Figs 2C and S5C. The core enriched genes are marked. (E) Comparison of gene expression of EMT-related genes in control and SAMD1 KO cells, based on RNA-Seq data. P-values are deried from DeSeq2. (F) RT-qPCR of EMT-related genes in BxPC3 or CFPAC cell lines comparing SAMD1 KO versus control cells. Data represent the mean ± SD of 3 biological replicates. Significance was analyzed using Student’s t test. (G) Expression of TJP1 in control and SAMD1 KO PaTu8998t cells based on RNA-Seq (upper panel) and immunofluorescence (lower panel). (H) Comparison of gene expression changes in PaTu8988t cells versus mouse ES cells [8] and HepG2 cells [19]. L3MBTL3 is the only gene that is consistely up-regulated. (I) Comparison of GSEA results from mesenchymal-related pathways in PaTu8988t, mouse ES [8], and HepG2 [19] cells upon SAMD1 KO. The data underlying this figure is available in S1 Data. (TIF) [file pbio.3002739.s005.tif]

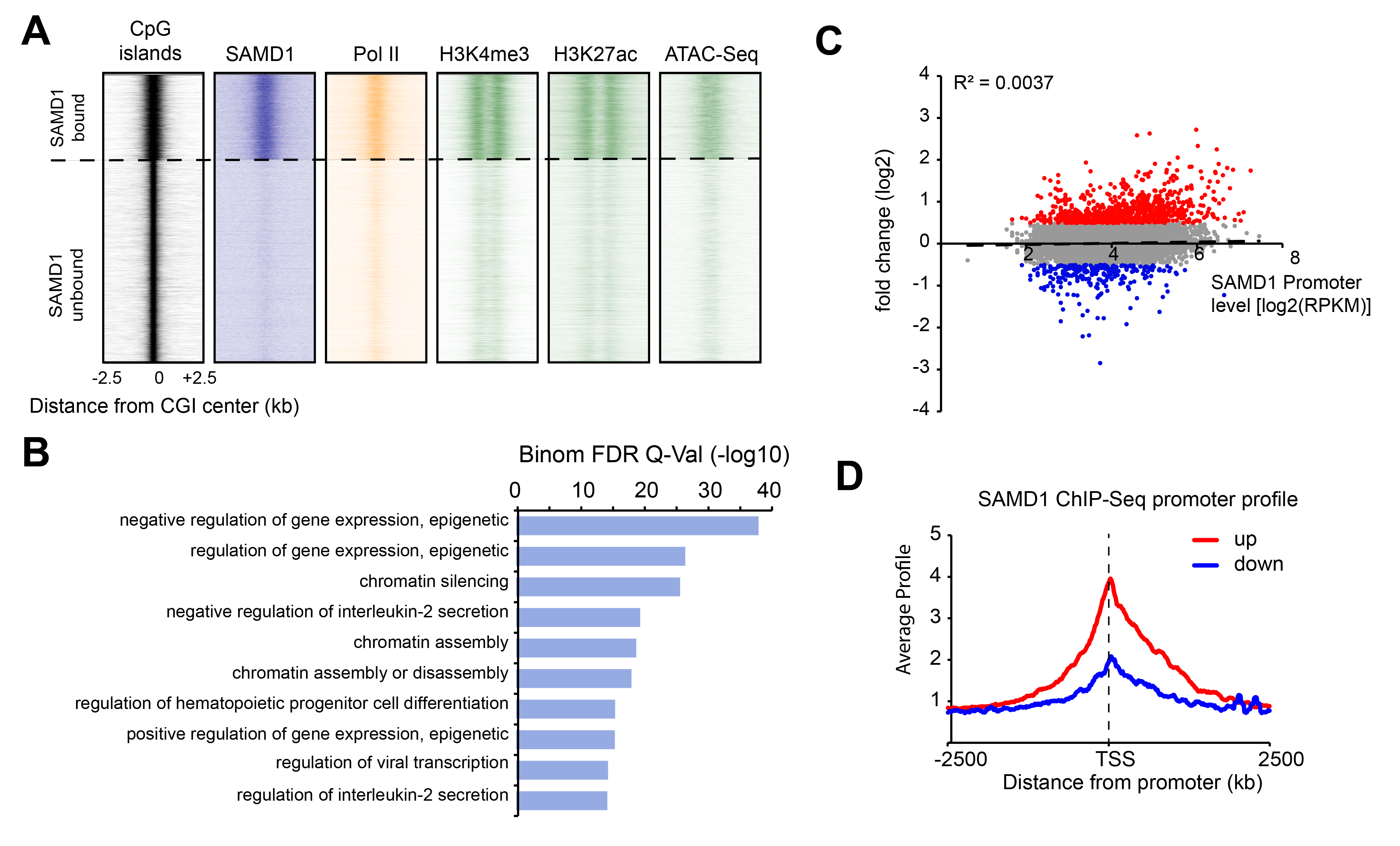

Supplement: S6 Fig — (A) Heatmaps of SAMD1-bound and -unbound CGIs regarding RNA Polymerase II, H3K4me3, H3K27ac, and ATAC-Seq. The heatmaps base on public ChIP-seq data, which can be found in the GEO database with accession numbers GSM945261, GSM818826, GSM1010788, and GSM1606403. (B) Gene ontology analysis of SAMD1 genomic targets using GREAT [54]. (C) Comparison of SAMD1 promoter level versus gene expression changes upon SAMD1 KO. Significant up- and down-regulated genes (cut-off: log2-fold-change > 0.5; p-value < 0.01) are colors in red and blue, respectively. (D) Promoter profiles of SAMD1 at the significant dysregulated genes from (C). The data underlying this figure is available in S1 Data. (TIF) [file pbio.3002739.s006.tif]

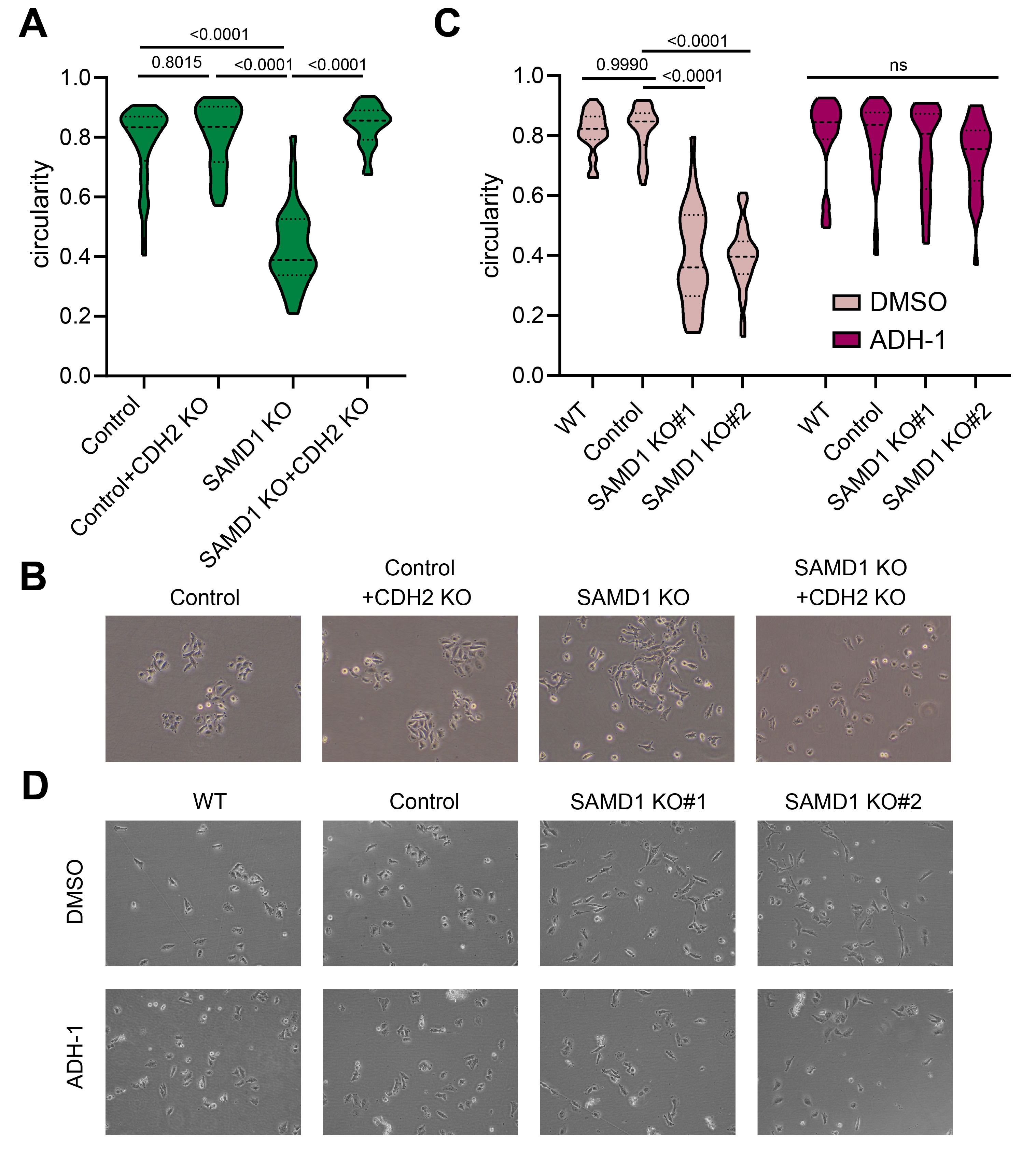

Supplement: S7 Fig — (A) Cell shape of control, CDH2 KO, SAMD1 KO, and CDH2/SAMD1 double KO PaTu8988t cells. Circularity was determined using ImageJ Fiji. Significance was analyzed using one-way ANOVA. (B) Example bright field microscopy for (A). (C) Cell shape of PaTu8988t wild-type cells, control cells, and 2 different SAMD1 knockout clones with or without application of the N-cadherin inhibitor ADH-1. Circularity was determined using ImageJ Fiji. Significance was analyzed using one-way ANOVA. (D) Example bright field microscopy for (C). The data underlying this figure is available in S1 Data. (TIF) [file pbio.3002739.s007.tif]

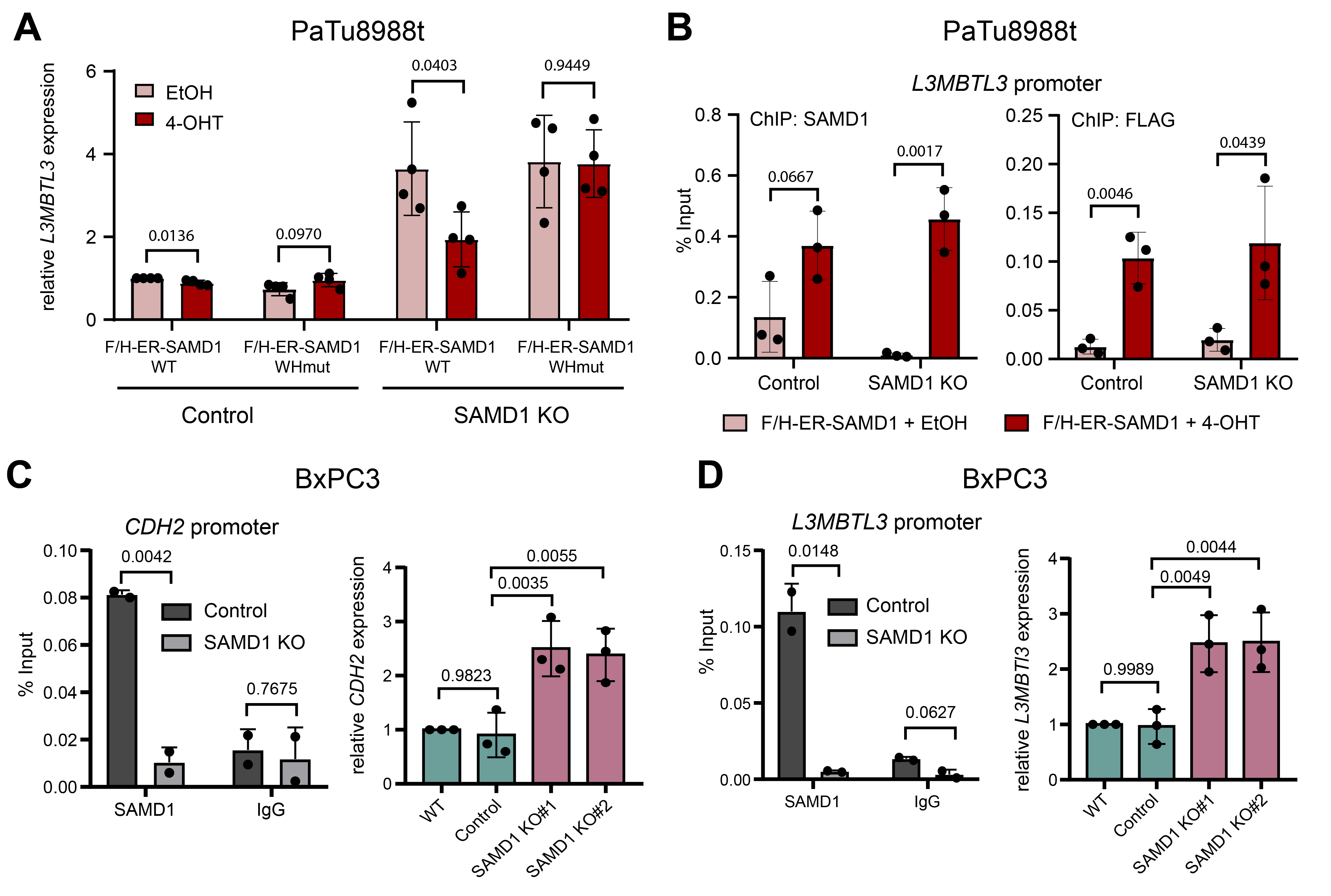

Supplement: S8 Fig — (A) RT-qPCR measuring L3MBTL3 expression with or without induction of SAMD1 rescue in PaTu8988t control and SAMD1 KO cells. WHmut = R45A/K46A mutation of SAMD1 [8]. Data represent the mean ± SD of 4 biological replicates. Significance was analyzed using Student’s t test. (B) SAMD1 ChIP-qPCR at the L3MBTL3 promoter with or without induction of SAMD1 rescue in PaTu8988t control and SAMD1 KO cells. Data represent the mean ± SD of 3 biological replicates. Significance was analyzed using Student’s t test. (C) ChIP-qPCR at the CDH2 promoter in BxPC3 control and SAMD1 KO cells using IgG or SAMD1 antibodies. Data represent the mean ± SD of 2 biological replicates. Significance was analyzed using Student’s t test. RT-qPCR measuring CDH2 expression in BxPC3 wild-type cells, control cells, and 2 different SAMD1 knockout clones. Data represent the mean ± SD of 3 biological replicates. Significance was analyzed using one-way ANOVA. (D) ChIP-qPCR at the L3MBTL3 promoter in BxPC3 control cells and SAMD1 KO cells, using SAMD1 or IgG antibodies. Data represent the mean ± SD of 2 biological replicates. Significance was analyzed using Student’s t test. RT-qPCR measuring L3MBTL3 expression in BxPC3 wild-type cells, control cells, and 2 different SAMD1 knockout clones. Data represent the mean ± SD of 3 biological replicates. Significance was analyzed using one-way ANOVA. The data underlying this figure is available in S1 Data. (TIF) [file pbio.3002739.s008.tif]

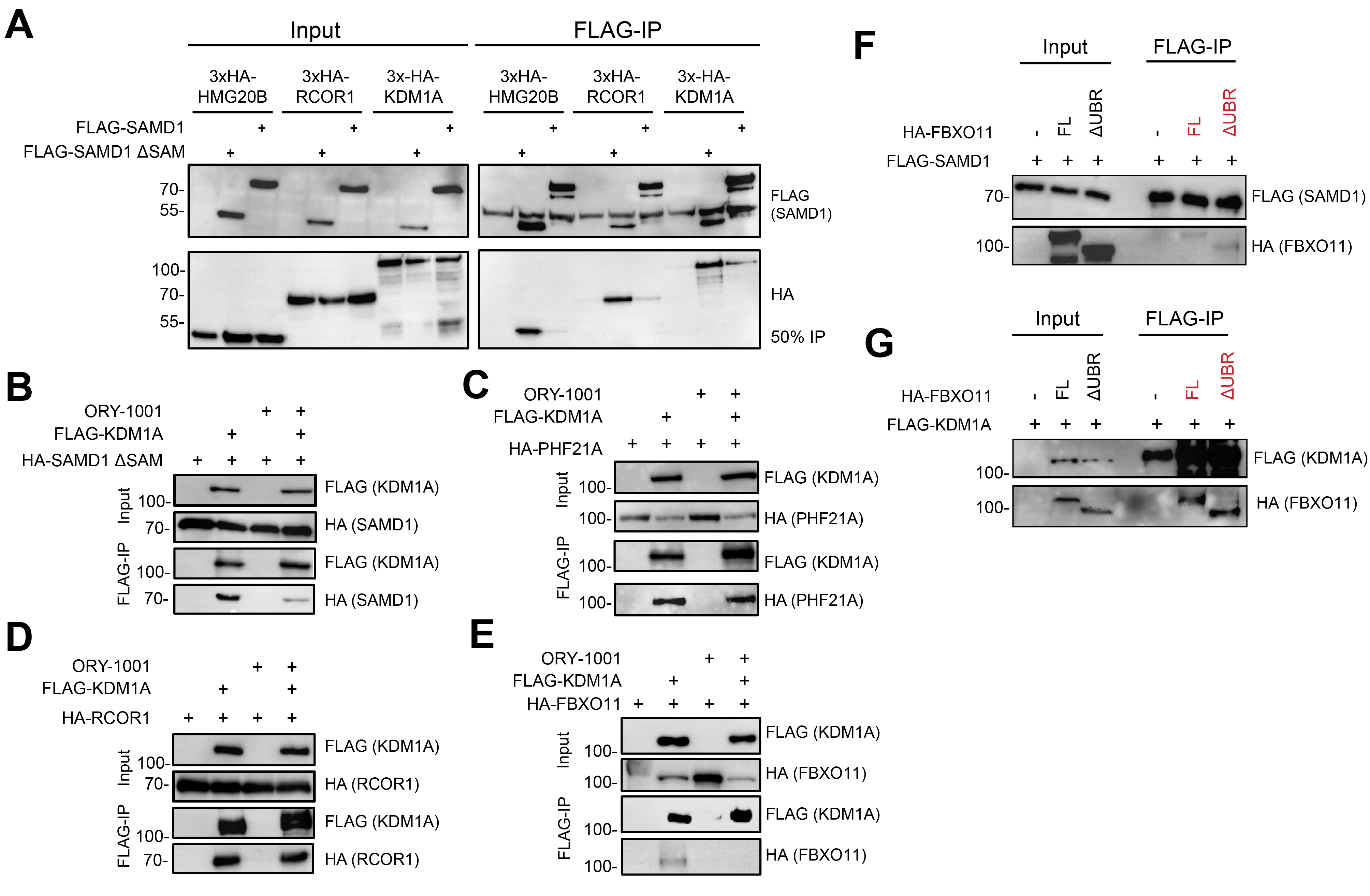

Supplement: S9 Fig — (A) Co-immunoprecipitation in HEK293 cells showing the interaction between SAMD1 full-length or SAMD1 ΔSAM and the KDM1A-complex. (B) Co-immunoprecipitation in HEK293 cells showing the interaction between SAMD1ΔSAM and KDM1A upon treatment with the KDM1A inhibitor ORY-1001. (C) Co-immunoprecipitation in HEK293 cells showing the interaction between PHF21A and KDM1A upon treatment with the KDM1A inhibitor ORY-1001. (D) Co-immunoprecipitation in HEK293 cells showing the interaction between RCOR1 and KDM1A upon treatment with the KDM1A inhibitor ORY-1001. (E) Co-immunoprecipitation in HEK293 cells showing the interaction between FBXO11 and KDM1A upon treatment with the KDM1A inhibitor ORY-1001. (F) Co-immunoprecipitation in HEK293 cells showing the interaction between full-length FBXO11 and FBXO11 ΔUBR with SAMD1. (G) Co-immunoprecipitation in HEK293 cells showing the interaction between full-length FBXO11 and FBXO11 ΔUBR with KDM1A. (TIF) [file pbio.3002739.s009.tif]

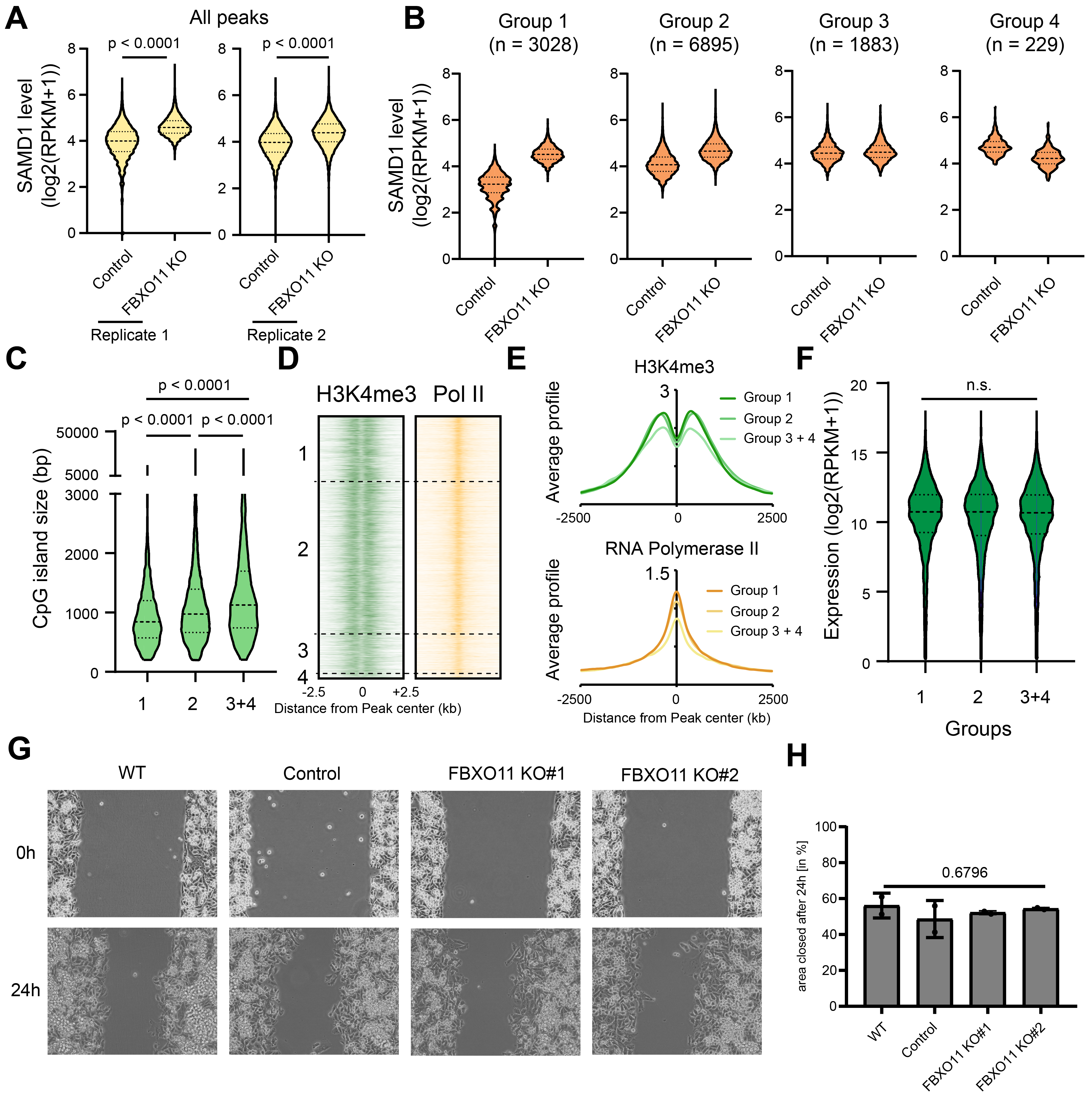

Supplement: S10 Fig — (A) Violin plots showing the SAMD1 level in PaTu8988t control and FBXO11 KO cells at SAMD1 bound locations for both ChIP-Seq replicates. Statistical significance was evaluated using a Kolmogorov—Smirnov test. (B) SAMD1 levels at the 4 different groups identified in Fig 6C in PaTu8988t control and FBXO11 KO cells (from replicate 1). (C) CpG island size of 4 different groups identified in Fig 6C. Statistical significance was evaluated using a Kolmogorov—Smirnov test. (D) Heatmap of H3K4me3 and Pol II in the 4 different groups identified in Fig 6C. The heatmaps base on public ChIP-seq data, which can be found in the GEO database with accession numbers GSM945261 and GSM1010788. (E) Profiles of H3K4me3 and RNA Polymerase II at the 4 different groups identified in Fig 6C. (F) Expression of genes in the 4 different groups identified in Fig 6C. (G) Representative picture of a wound healing assay of PaTu8988t wild-type cells, control cells, and 2 different FBXO11 knockout clones. (H) Quantification of (G). Data represent the mean ± SD of 2 biological replicates. Significance evaluated using one-way ANOVA. The data underlying this figure is available in S1 Data. (TIF) [file pbio.3002739.s010.tif]

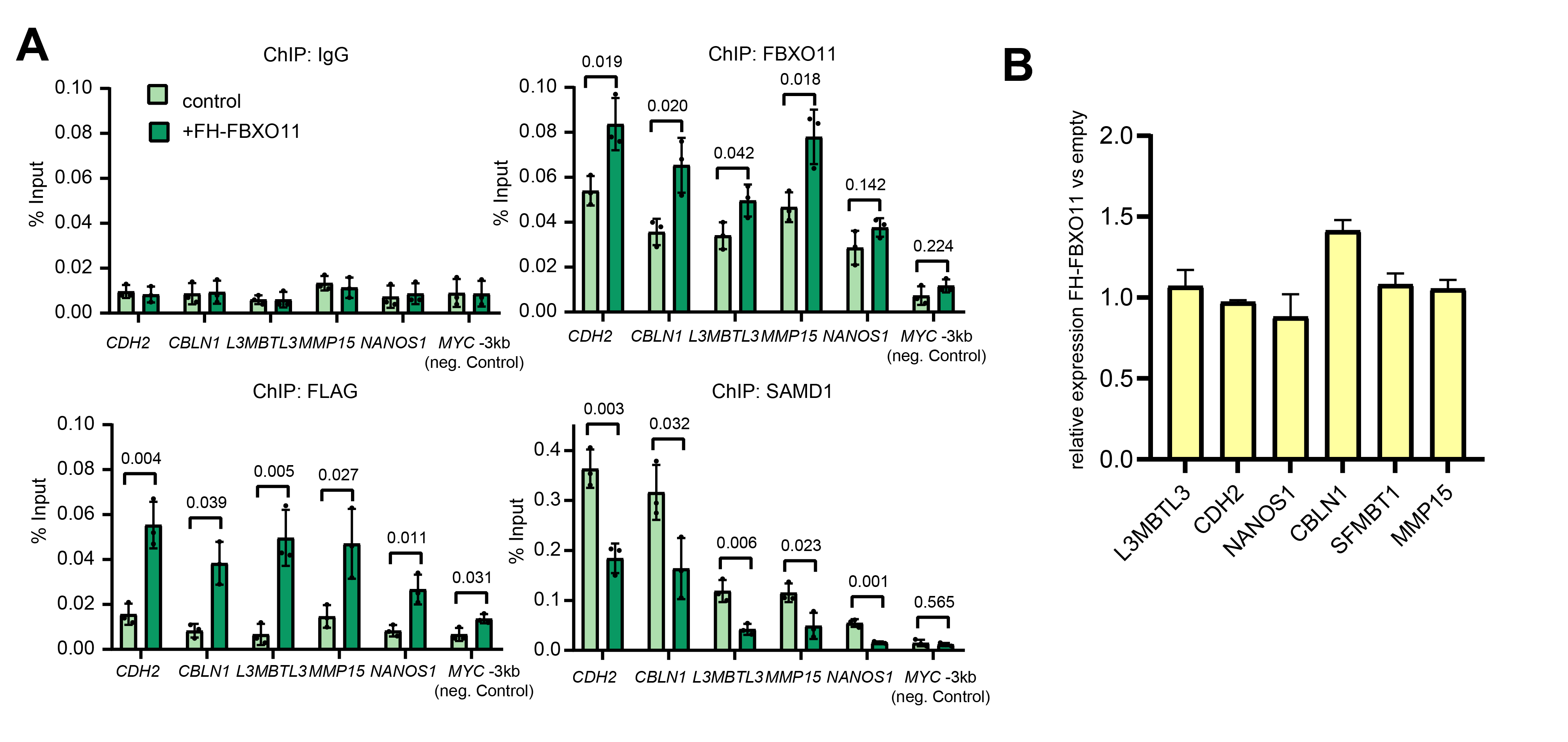

Supplement: S11 Fig — (A) ChIP-qPCR of FBXO11 and SAMD1 after FBXO11 overexpression in HEK293 cells. Data represent the mean ± SD of 3 biological replicates. Significance was analyzed using Student’s t test. (B) Relative expression of SAMD1 target genes from (A) upon FBXO11 overexpression measured by RT-qPCR. Data represent the mean ± SD of 3 biological replicates. The data underlying this figure is available in S1 Data. (TIF) [file pbio.3002739.s011.tif]

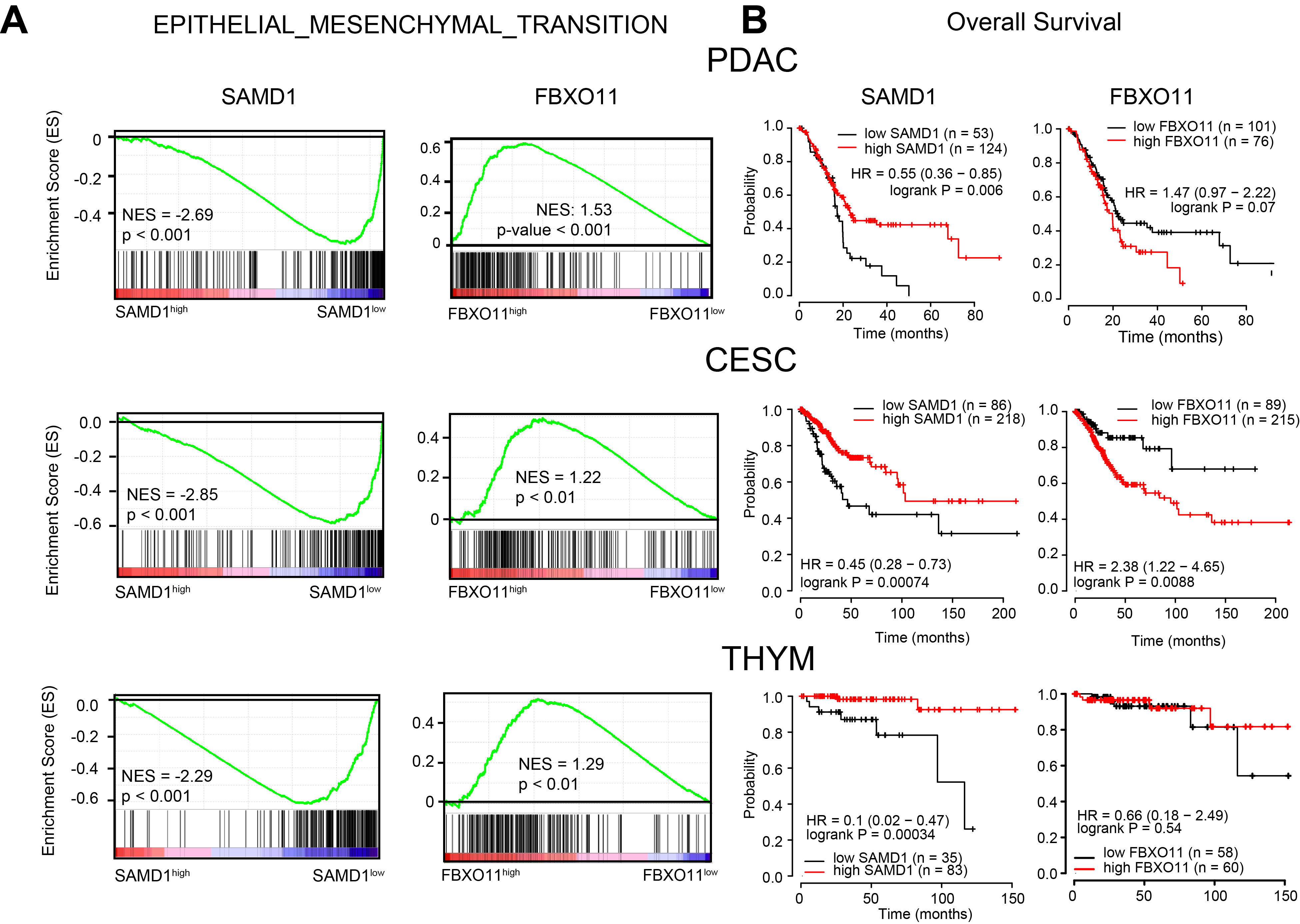

Supplement: S12 Fig — (A) GSEA of the epithelial—Mesenchymal transition (EMT) pathway comparing patient samples with SAMD1 high versus SAMD1 low and FBXO11 high versus FBXO11 low. (B) Kaplan—Meier survival curves (overall survival) based on SAMD1 and FBXO11 expression. Data are derived from TCGA and visualized via the Kaplan—Meier plotter tool [22] using auto-selected cut-off. Some of the results are already shown in Figs 1, 6 and S1, but are included here for comparison. The data underlying this figure is available in S1 Data. (TIF) [file pbio.3002739.s012.tif]
