# Supplementary material for: SAMD1 suppresses epithelial–mesenchymal transition pathways in pancreatic ductal adenocarcinoma
Source: PLoS Biol. 2024 Aug 13;22(8):e3002739. doi: 10.1371/journal.pbio.3002739 (PMC11343471; doi:10.1371/journal.pbio.3002739)

S12 Fig. Raw images

Figure 1C

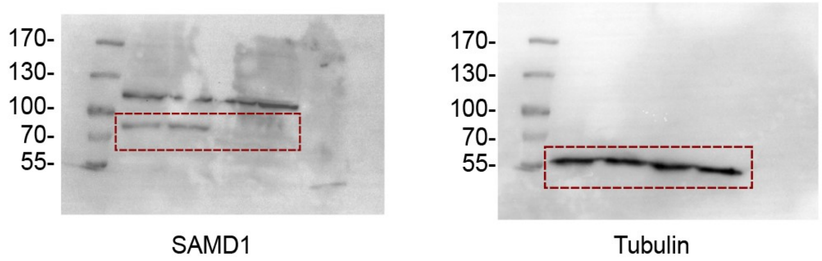

Figure 3C

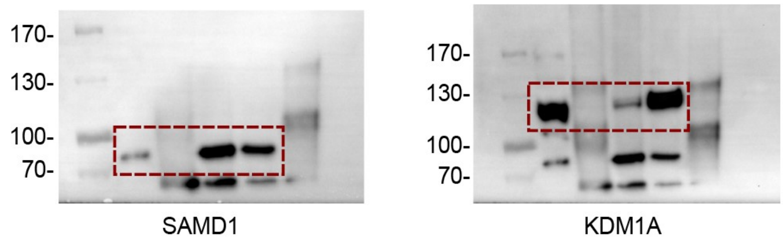

Figure 3E

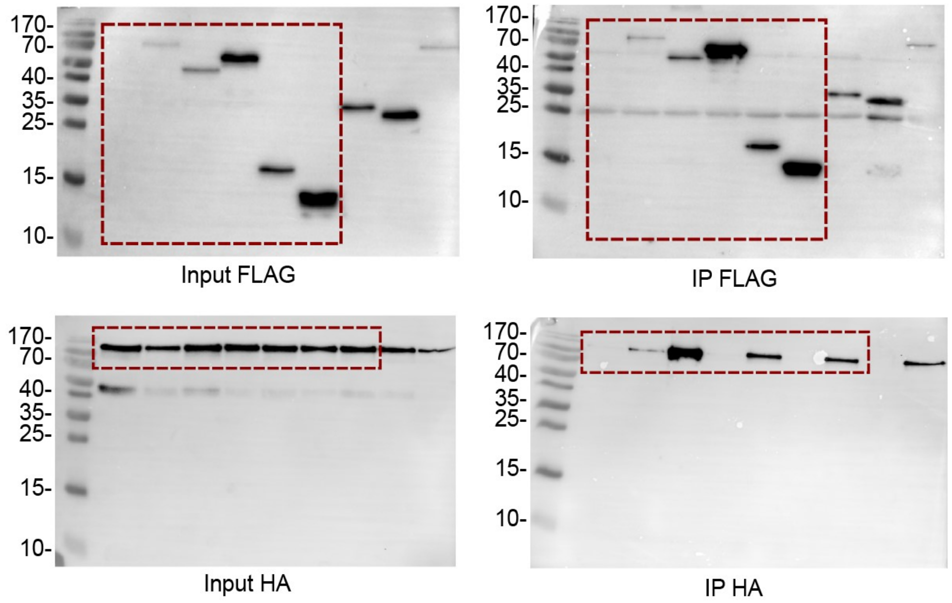

Figure 3F (left)

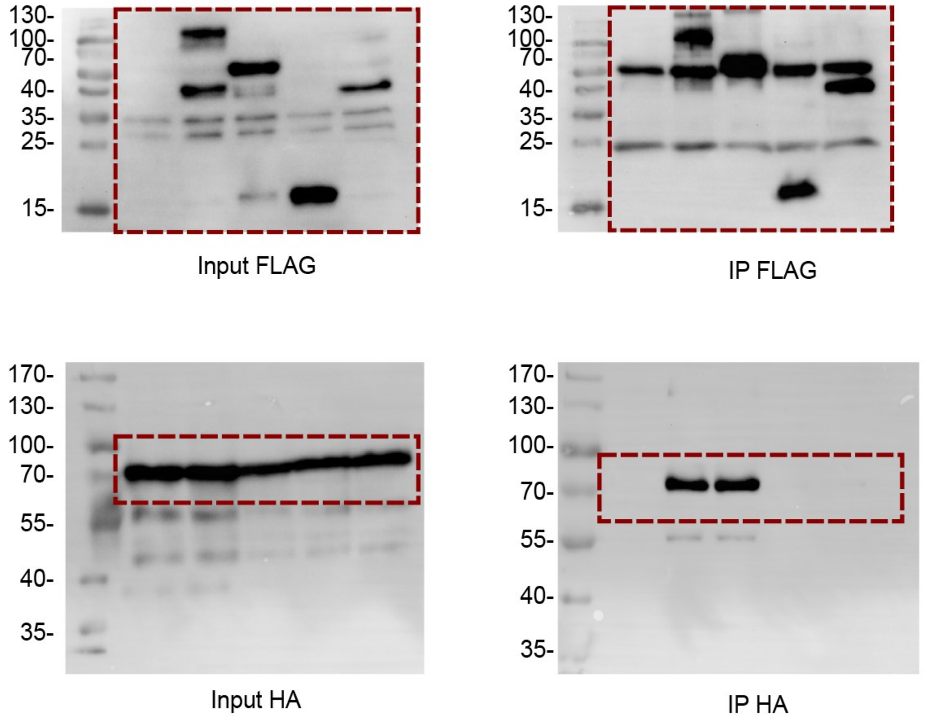

Figure 3F (right)

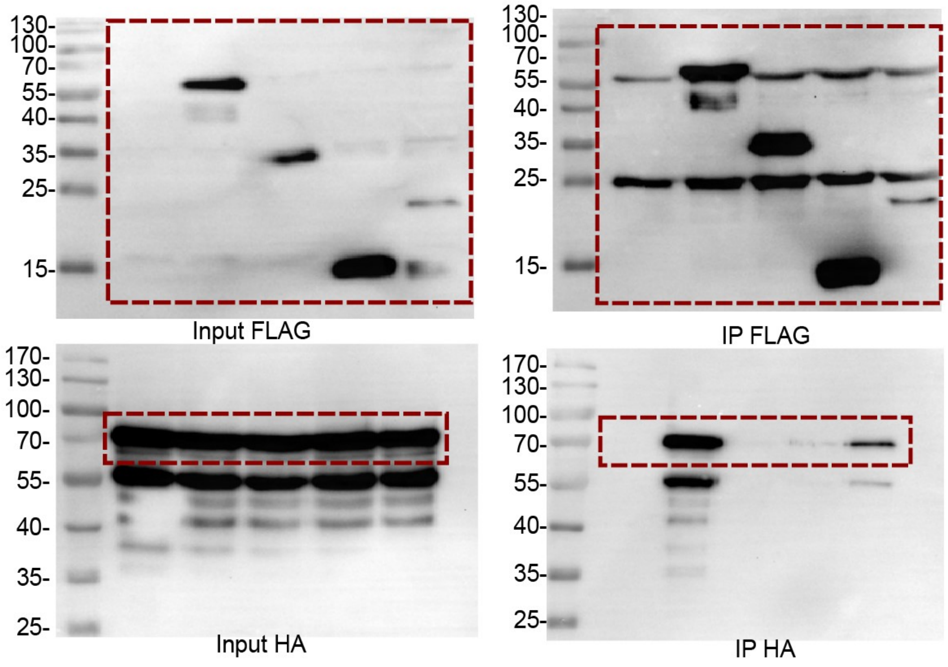

Figure 3G

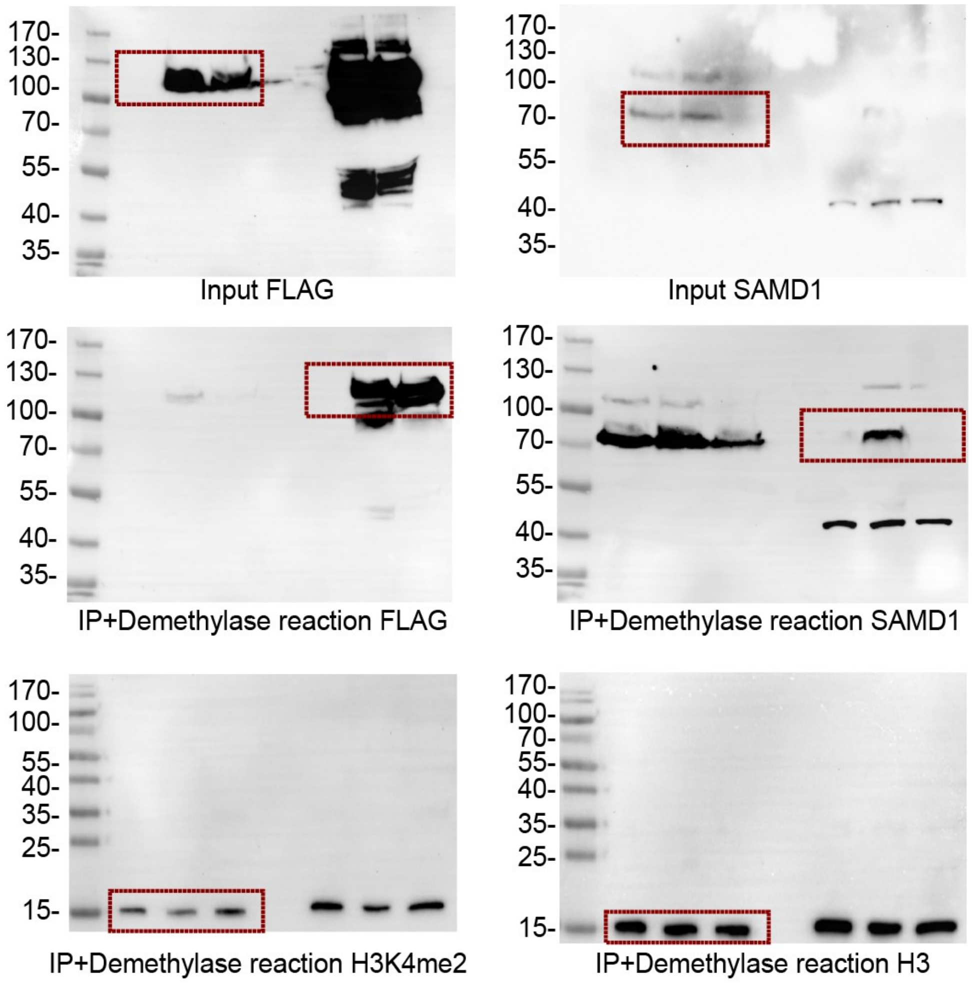
170-
130-
100-
70-
55-
40-
35-
Input FLAG
Input SAMD1
IP+Demethylase reaction FLAG
IP+Demethylase reaction SAMD1
25-
15-
IP+Demethylase reaction H3K4me2
IP+Demethylase reaction H3

Figure 4A

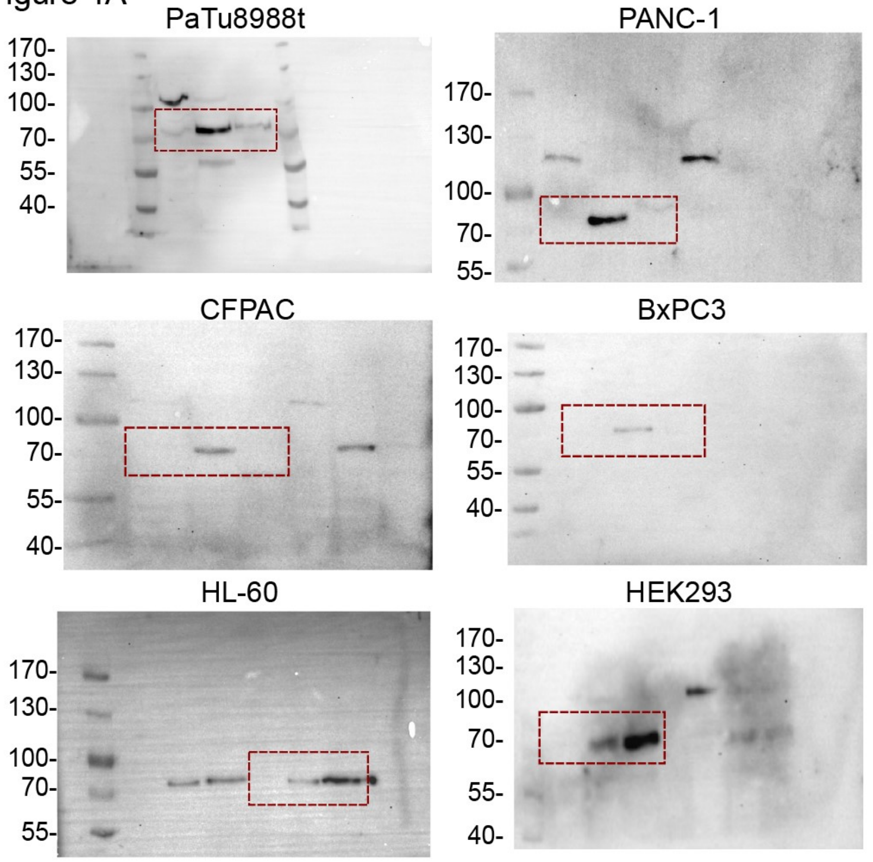

Figure 4D

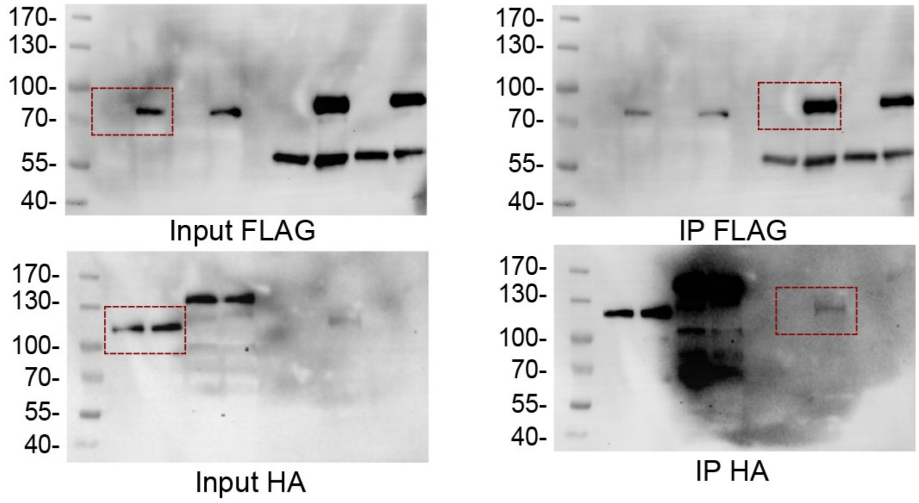

Figure 4E

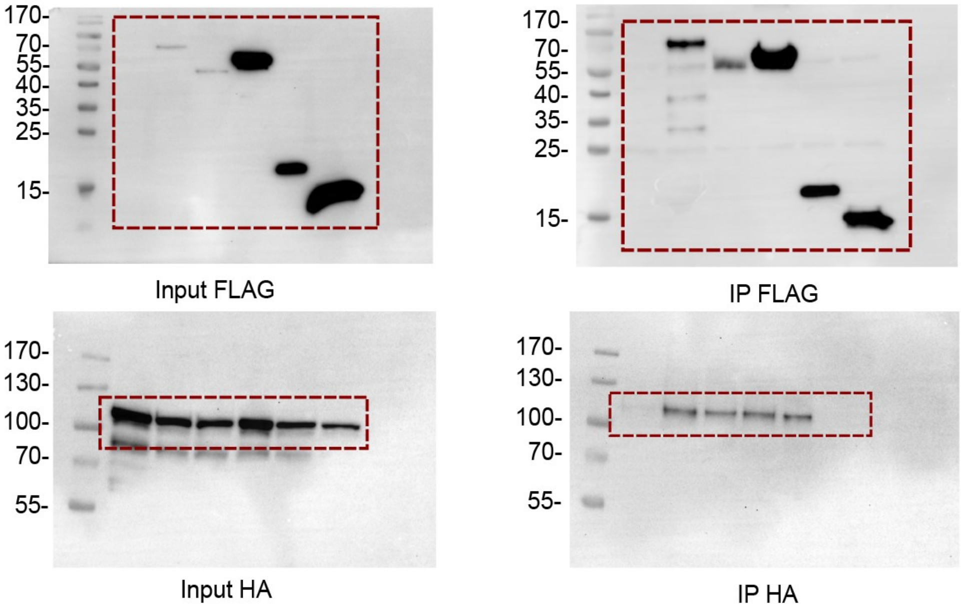

Figure 4F (left)

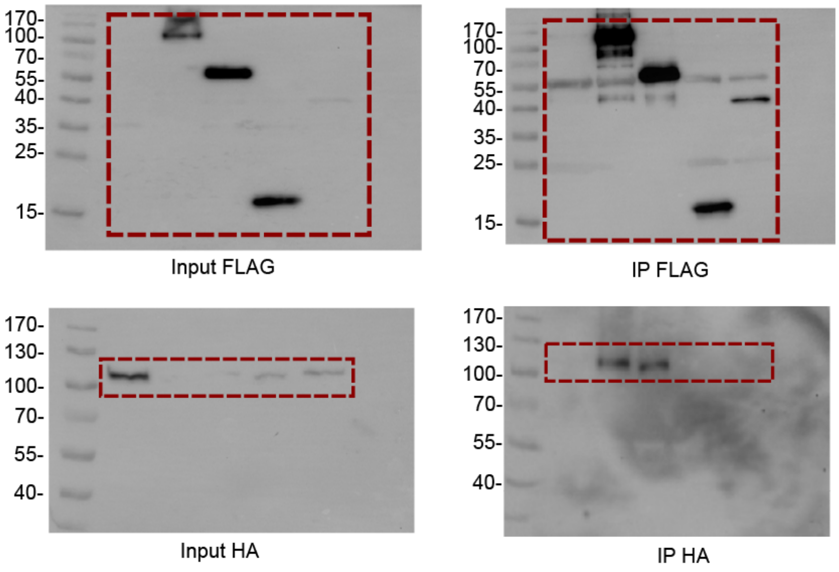

Figure 4G (right)

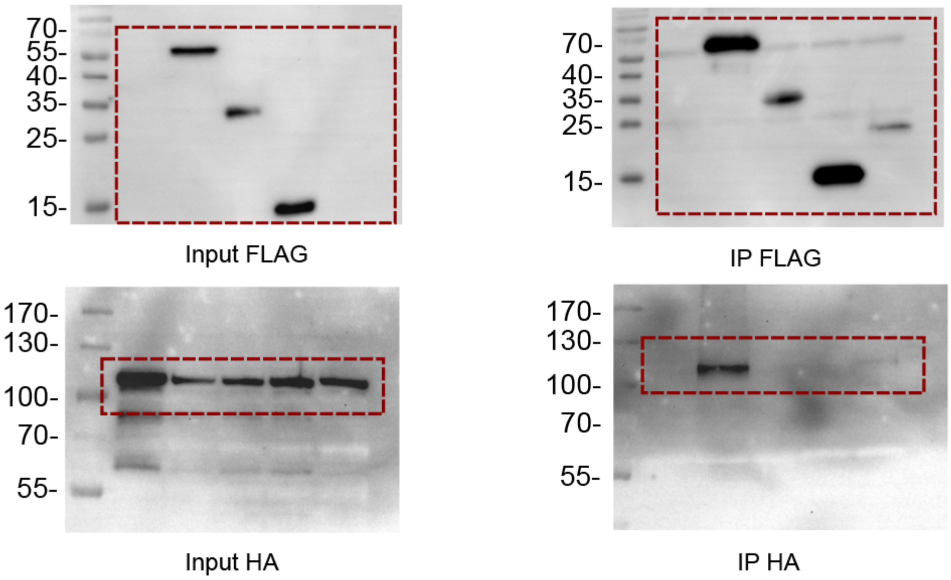

Figure 4G

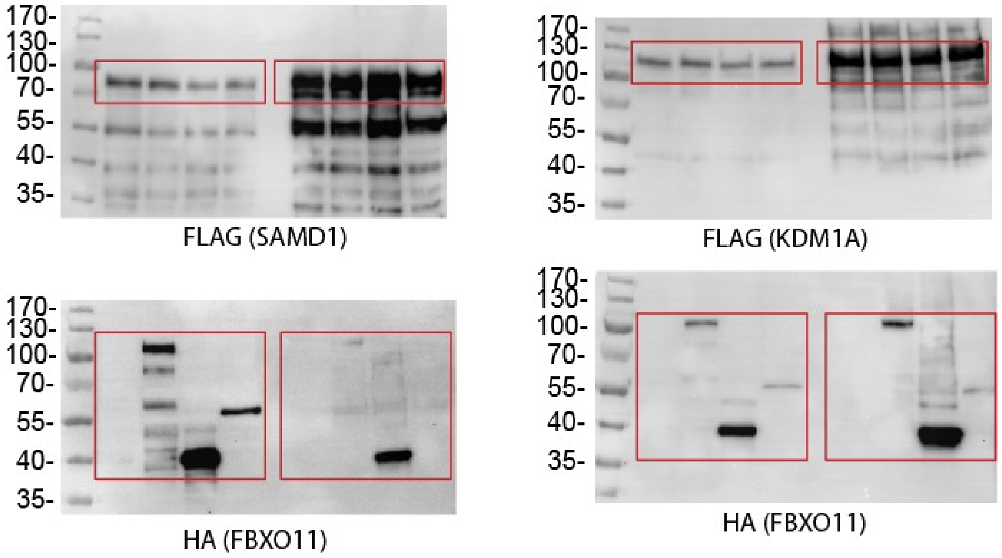

Figure 4H

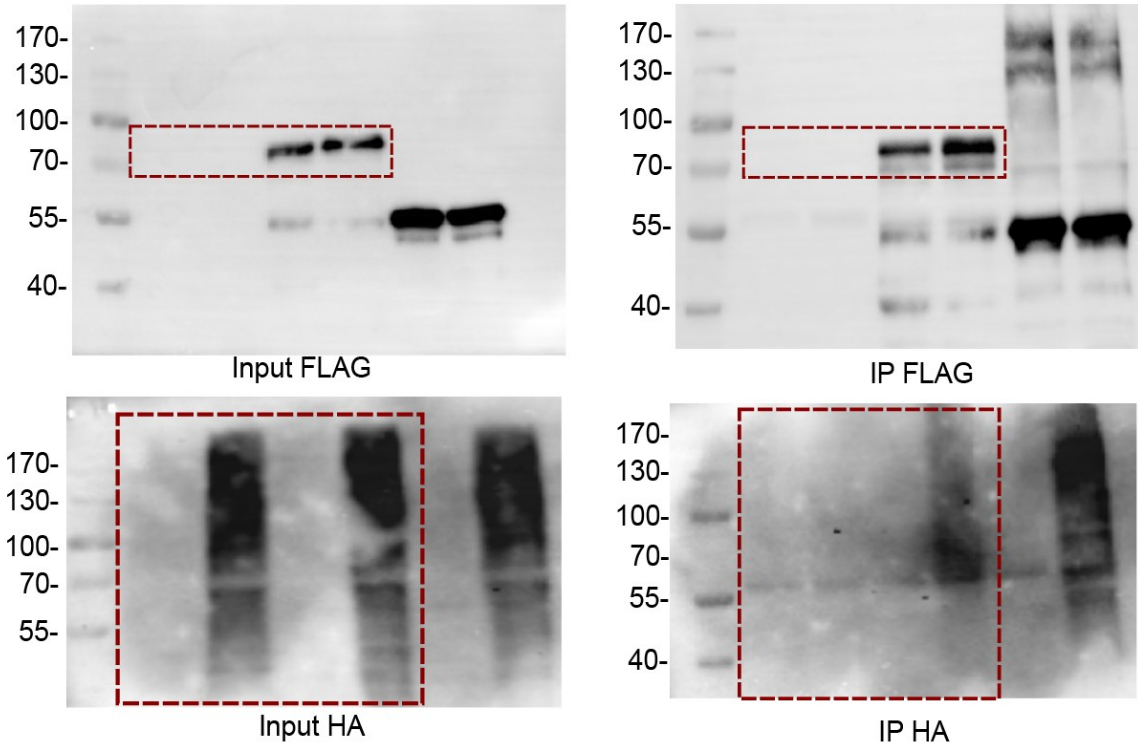

Figure 4I

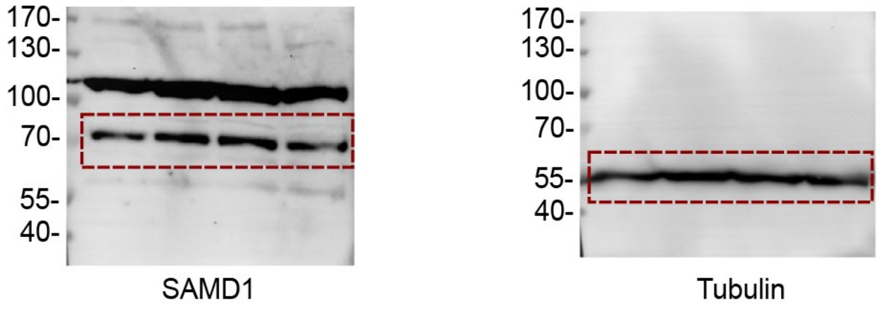

Figure 5A

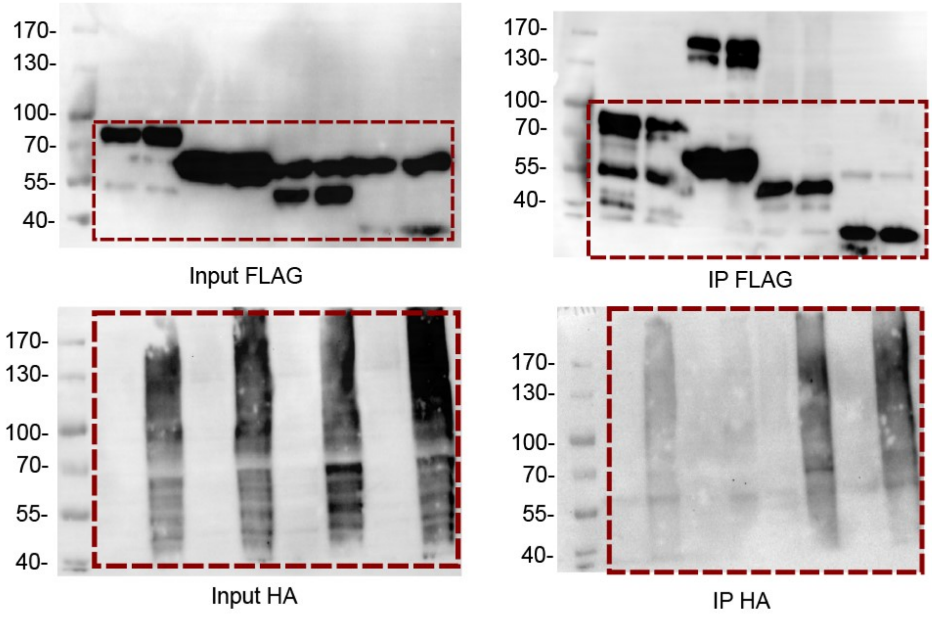

Figure 5B

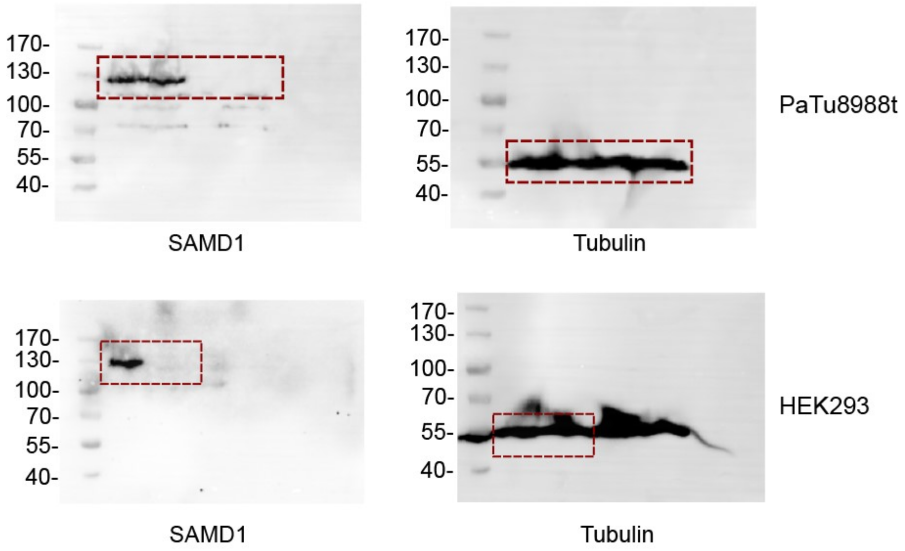

Figure 5C

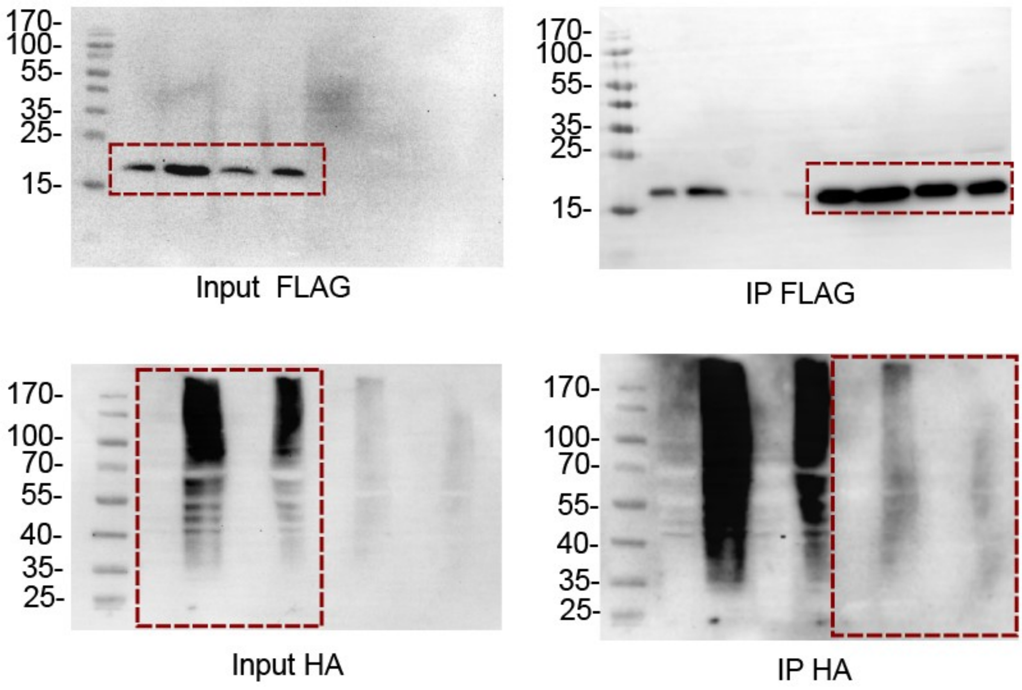

Figure 5E

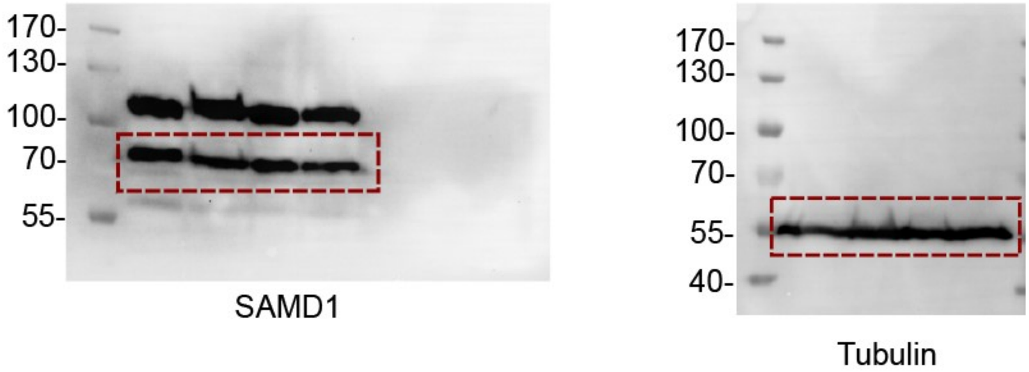

Figure 5F

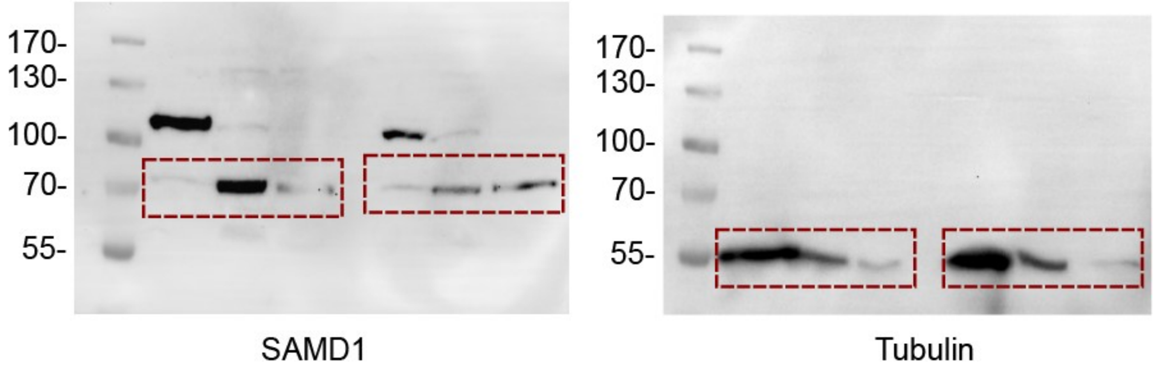

Figure S3A

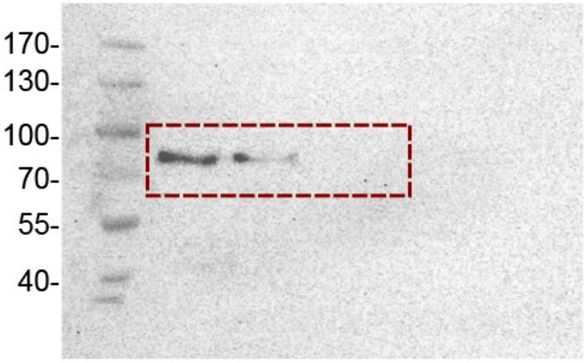

SAMD1

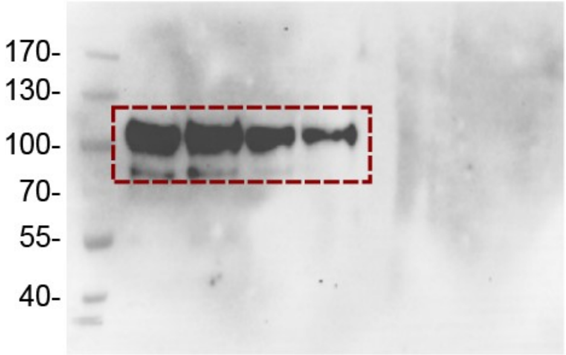

SP1

Figure S4B

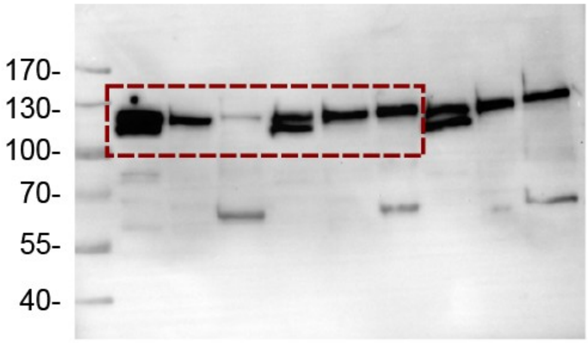

SAMD1

Figure S9A

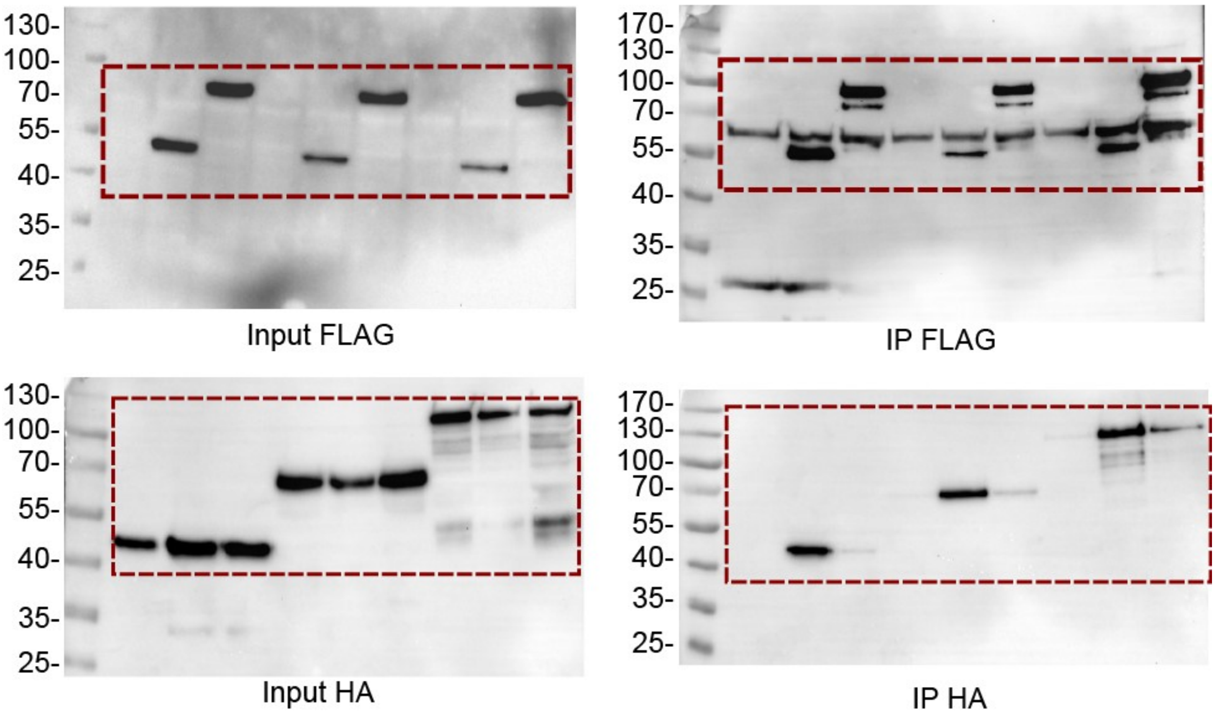

Figure S9B

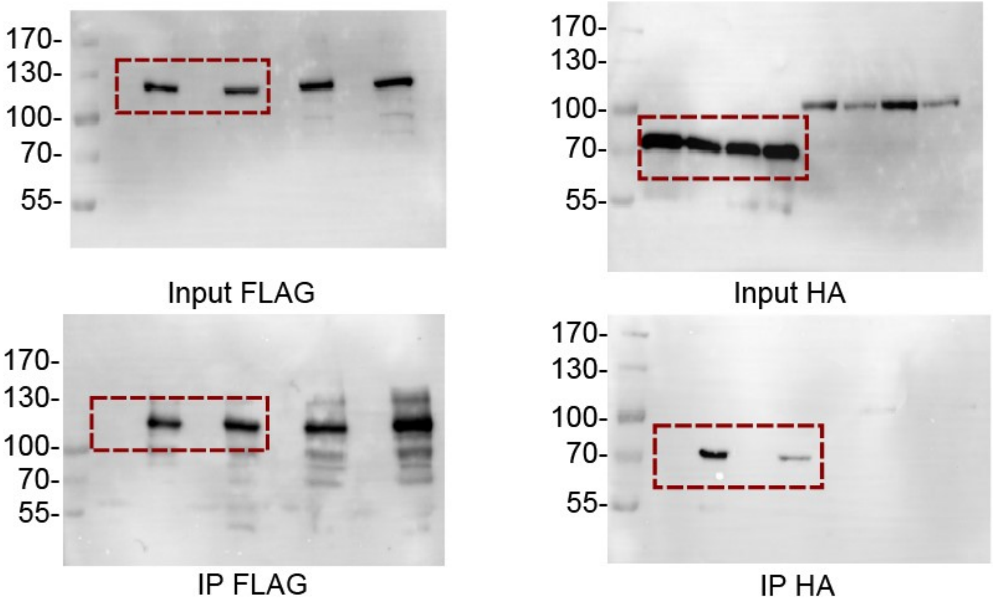

Figure S9C

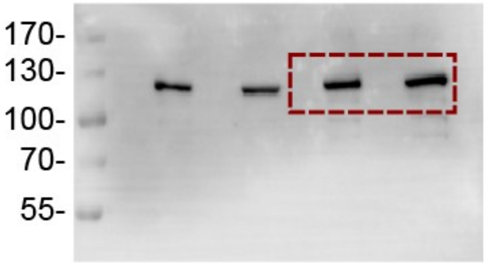

Input FLAG

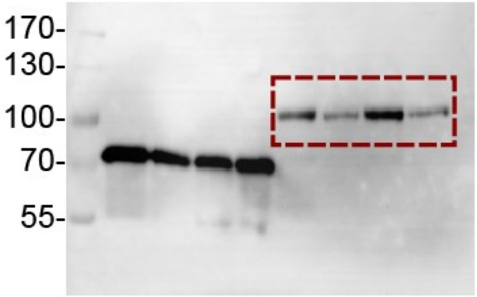

Input HA

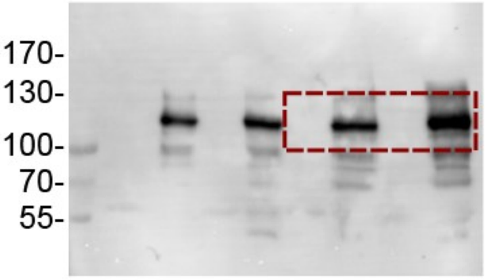

IP FLAG

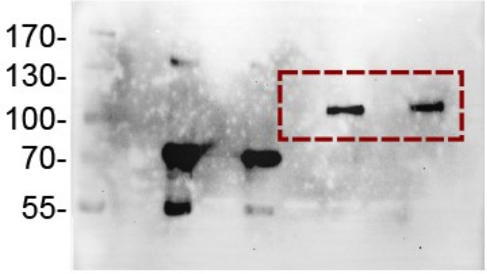

IP HA

Figure S9D

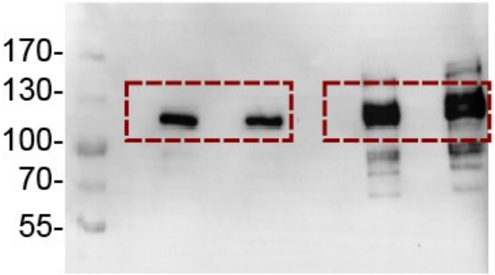

Input + IP FLAG

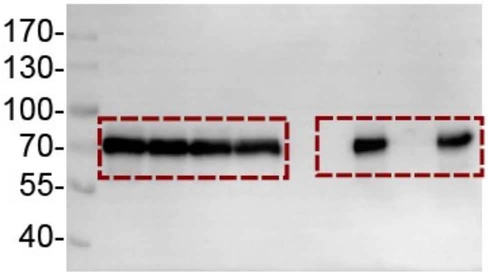

Input + IP HA

Figure S9E

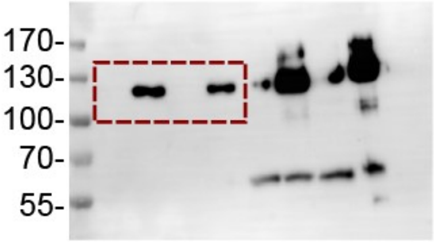

Input FLAG

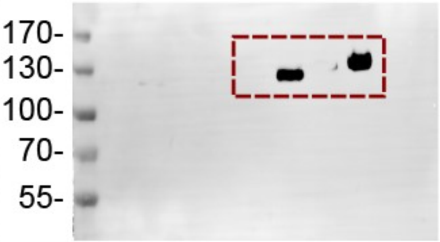

IP FLAG

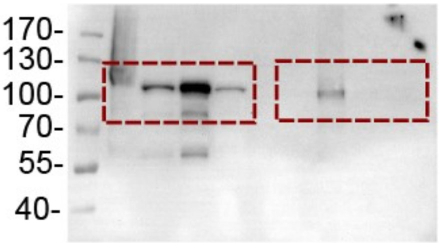

Input + IP HA

Figure S9F

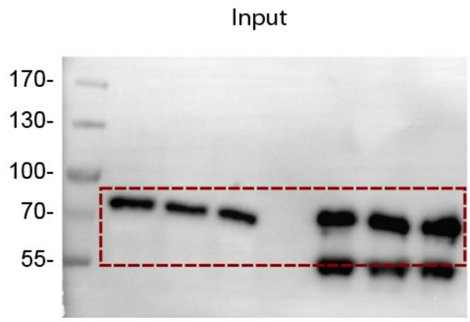

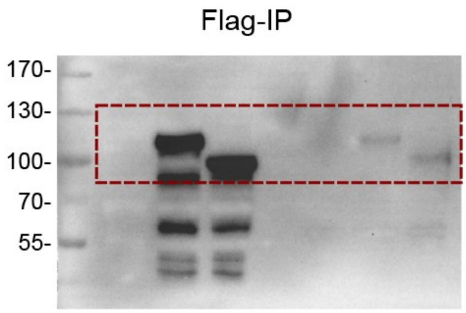

Figure S9G

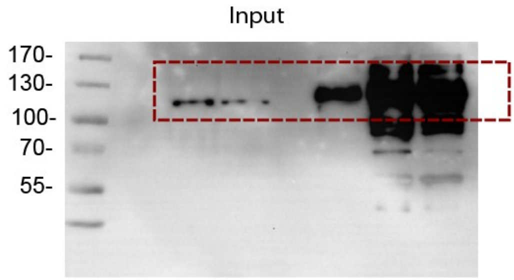

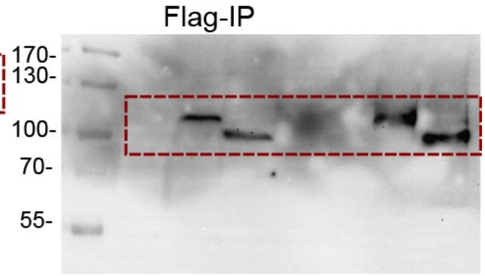

Supplement: S1 Raw Images — (PDF) [file pbio.3002739.s018.pdf]
