# Supplementary material for: SAMD1 suppresses epithelial–mesenchymal transition pathways in pancreatic ductal adenocarcinoma
Source: PLoS Biol. 2024 Aug 13;22(8):e3002739. doi: 10.1371/journal.pbio.3002739 (PMC11343471; doi:10.1371/journal.pbio.3002739)
Supplement: Attachment — Submitted filename: Response to Reviewers.pdf [file pbio.3002739.s019.pdf]

We thank all reviewers for their insightful and constructive comments. We made the following major changes to the manuscript:
(The line numbers refer to the revised manuscript without track changes)

1. We performed additional biological assays that confirmed the EMT phenotype of the SAMD1 KO cells, such as adhesion and invasion assays (Figure S2).
2. We emphasize now that besides *CDH2* many other EMT-related genes are upregulated (Lines 200-202). In addition, we also show the upregulation of other mesenchymal pathways (Figure S5C, D).
3. We have included more bioinformatic analysis which showed that SAMD1 binds to active CpG islands, which are enriched for CGCG motifs (Figure 2E, S6A). This result is consistent with previous results from our lab.
4. We show that FBXO11 overexpression leads to the reduction of SAMD1 chromatin binding, which is the opposite of the consequences of the knockout (Figure S11). This result confirms our hypothesis that FBXO11 is a negative regulator for the chromatin binding of SAMD1.
5. We have improved the Co-Immunoprecipitation experiments. (Figure 3F).
6. We have improved the manuscript text to make some points clearer.
7. We have adapted the manuscript to the PLOS Biology Style.
8. We now include all raw data as supplementary data files.

Other changes are outlined below.

We hope these changes can convince the reviewers about the quality and validity of our work.

Reviews

Reviewer #1: In the manuscript by Simon et al. the authors addressed the role of the CpG island binding protein SAMD1 in pancreatic cancer. They find that it is upregulated in several cancers, but displays potentially opposing roles in cancer as its expression either correlates or anticorrelates with prognosis. In PDAC SAMD1 was found to repress genes associated with epithelial-mesenchymal transition (like N-cadherin) and knockout of SAMD1 in PaTu8988t and BxPC3 cell lines resulted in derepression to unleash migration. The authors found that SAMD1 function depends on its interaction with KDM1A and FBXO11, suggested to form a ternary complex that is recruited to CpG islands at target promoters.
We lack of sufficient mechanistic insight how deregulated gene expression rapidly renders malignant transformation and therapy resistance. Hence, identifying exploitable vulnerabilities is of high relevance. Therefore, the manuscript is of potential interest and merits to be published in PLoS Biology. The analyses are strong in dissecting the mechanism how SAMD1 is interacting with cofactors and how this impacts regulation of target genes. In particular, which protein domains of SAMD1, FBXO11 and KMD1A are involved in binding was done very accurately. Where the story falls short is in understanding how SAMD1 can have context-dependent protumorigenic and antitumorigenic functions. Also the analysis of the role of the SAMD1-FBXO11 interaction is a bit descriptive only. This should be improved in a revised version. Until now, the FBXO11 part is a bit isolated and the consequence of FBXO11 loss remains unknown. I listed my specific comments below:

We thank the reviewer for the positive and constructive comments about our manuscript. Please find below responses to specific questions.

1. Repression of CDH2 is a main finding to enable EMT and cell motitity. However, mechanistic dissection revealed that in the end KO of FBXO11, as novel interaction partner of SAMD1, was not so much affecting CDH2 expression (and of the bona fide target L3MBTL3), although FBXO11 KO results in global increase of SAMD1 chromatin binding and in particular at the CDH2 promoter. So there is something puzzling that does not really fit. Based on the model in Fig. 6i,j the complexes are mutually exclusive, which the data do not support (in fact more the opposite?!). Even if there are different complexes, the FBXO11 function does not affect any of the previously identified targets. The only link between SAMD1 and FBXO11 function is that the EMT score of FBXO11-hi vs. low tumor samples from TCGA in Fig. 6h is increased in FBXO11-hi tumors, which might be completely independent from SAMD1.

The reviewer addresses a good point. Indeed, the effect of FBXO11 on the function of SAMD1 regarding EMT appears to be subtle, given that *CDH2* does not become downregulated upon FBXO11 KO. The likely explanation is that a 50% increase in SAMD1 levels at the *CDH2* promoter is not sufficient to decrease the expression of *CDH2*. Perhaps this subtle change of SAMD1 levels at the promoter can be compensated by other regulatory mechanisms. This is different from the complete absence of SAMD1 in the SAMD1 KO cells, which perhaps can not be compensated by the transcription machinery.

We speculated that the overexpression of FBXO11 may lead to better results. Unfortunately, overexpression of FBXO11 was not possible in PaTu8988 cells, because this led to cell death. A similar problem we had before for SAMD1 itself.

For the revision, we attempted two things:

1) We used HEK293 cells as an alternative cellular model where FBXO11 overexpression was possible. In this context, we could show that an increase in FBXO11 levels leads to a reduced chromatin binding of SAMD1. This is consistent with our working hypothesis. Notably, in this experiment, we also could not observe strong changes in gene expression suggesting that the 50% reduction of SAMD1 on the target genes has only minor consequences (see Figure S11 A and B).
2) We cloned FBXO11 with an ER-fusion protein and performed a similar experiment as before for SAMD1. After induction of ER-FBXO11 with tamoxifen, we could demonstrate the SAMD1 chromatin level becomes somewhat lower (see below). However, again we could not observe strong changes in gene expression. We decided to not include this result in the manuscript, given that the effects are not significant.

Altogether these experiments support our hypothesis that FBXO11 is an important regulator of SAMD1 chromatin binding, but the effect regarding gene expression appears to be more subtle than originally anticipated. We have now discussed this aspect in more detail in the results part and the discussion (lines 482-486, 495-498, 586-589).

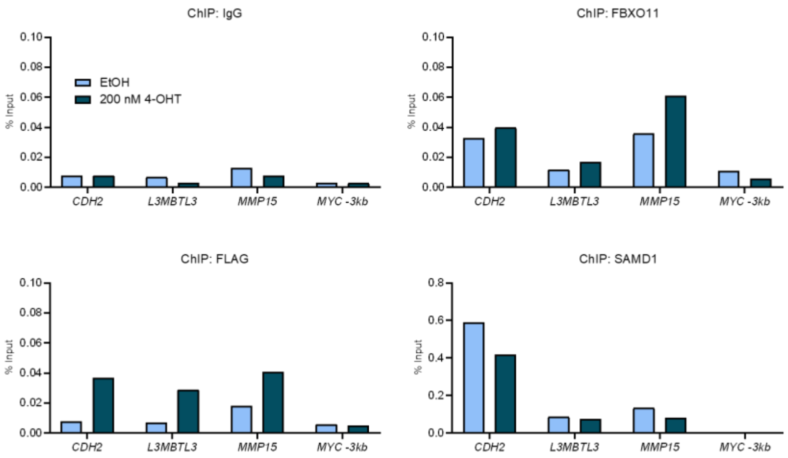

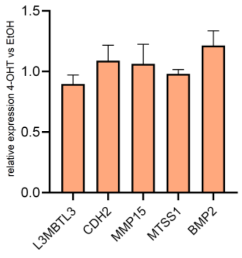

Of note, a recent publication, which analyzes histone-binding proteins via mass spectrometry, supports an opposing role of SAMD1 and FBXO11 on chromatin (PMID: 38448585). In the respective database (https://marcs.helmholtz-munich.de/readers/dna_methylation) SAMD1 is shown to be repelled by methylated DNA, consistent with our previous work. In contrast, FBXO11 was found to be attracted by methylated DNA. This unexpected finding (so far, no chromatin binding of FBXO11 has been described), suggests that FBXO11 is recruited to methylated genomic regions, where it may influence other chromatin-binding proteins, including SAMD1.

Given that SAMD1, FBXO11, and KDM1A all interact with each other, we do not think these complexes are mutually exclusive, as our original model in 6J may have implied. Thus, we have now modified the model in Figure 6J, to clearly point out that FBXO11 deletion leads to enhanced chromatin binding of SAMD1, and that FBXO11 can bind to SAMD1 also when KDM1A is bound.

2. In line with that, the statement in lines 495-499 "…types, such as diffuse large B-cell lymphomas and lung cancer [31,34]. In lung cancer cell lines, the FBXO11-containing complex was found to neddylate p53, thereby inhibiting its transcriptional activity." suggests that the functional connection of SAMD1 and FBXO11 cannot be clearly separated from other independent functions of FBXO11. Likely, there are overlapping effects and apparently the SAMD1 specific effect of FBXO11 is minor. Moreover, since the PaTu8988t cell line is mutant for p53, loss or gain of function of mutant neddylated p53 or effects of FBXO11/p53 binding could mainly influence the cellular phenotype, independent of SAMD1.

The reviewer is absolutely right, that FBXO11 likely has many other functions not related to SAMD1. Consequently, the effect of FBXO11 on SAMD1 may only be a minor aspect of the overall function of FBXO11. Nonetheless, our work identified FBXO11 as a novel regulatory partner of SAMD1, which expands the repertoire of functions of FBXO11. We are confident that this is a piece of important information for future research on both FBXO11 and SAMD1. In the revised manuscript, we also show now that FBXO11 overexpression in HEK293 cells leads to a reduced chromatin binding of SAMD1, consistent with our hypothesis (see Figure

S11A). In addition, our experiment suggests that FBXO11 may bind to chromatin, which could be of interest for future studies on FBXO11, as well.
We have now improved the discussion (lines 545-555), to emphasize that FBXO11 has many more functions, beyond its role on SAMD1.

3. Fig. 3b,4f: There is no detectable expression of Tower and AOD_C in input detected and the IP is also not convincing for these fragments, so it is difficult to draw robust conclusions from this experiment. Especially in the 'Tower sample: Which of the many bands in the IP is actually the IP'd protein? This needs to be improved. Similarly, in lines 337-339: "This interaction is facilitated by the N-terminal part of the AOD domain of KDM1A, which is the same region as for the interaction with SAMD1 (Figure 4f, 3f)." This conclusion is not very convincing, since AOD_C and AOD_N are hardly expressed or IP'd. So the findings should be confirmed by a SAMD1delAOC-N vs. SAMD1delAOC-C construct.

We repeated the experiment for Figure 3F and were able to improve the results. Tower and AOD-C are now clearly detectable. We agree that the N-terminal part of the amino oxidase domain (AOD-N) is weakly expressed (and therefore difficult to detect in the input samples, but clearly visible in the IP), but still we can detect an interaction with SAMD1 and FBXO11 (Figure 3F and 4F, right part), demonstrating that a small amount is already sufficient for the interaction, which makes it even more convincing.
We additionally cloned constructs with deletions of either the N-terminal or the C-terminal region of KDM1A's amino oxidase domain. However, both constructs still interact with SAMD1 and FBXO11 in Co-IP experiments. This result is not unexpected as we have demonstrated that SAMD1 also strongly interacts with other components of the KDM1A complex, such as RCOR1 and HMG20B (see Figure S9 A). Due to these results, we can conclude that the AOD-N is involved, but not essential for the association between SAMD1 and the KDM1A complex. The text was now modified to state that the AOD-N region is involved in the interaction with both FBXO11 and SAMD1, but we do not state anymore that this is the only interacting region. (lines 383-387)

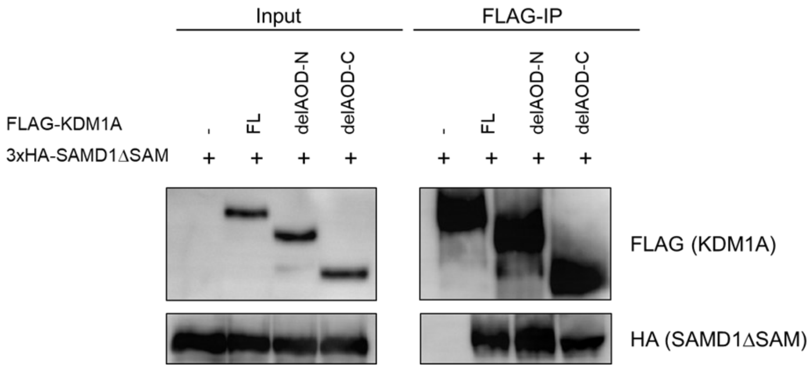

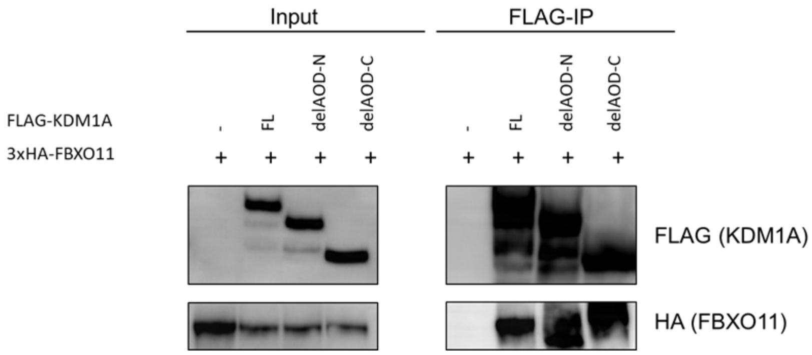

An additional point: I can see the non-cropped immunoblots in the supplement with MW indicators, but in particular for the IPs with the fragments of different size, MW indicators would be helpful.

We thank the reviewer for pointing this out. We now included molecular weight indicators for all western blots.

4. Lines 283-285, Fig. 3g,h: "This result suggests that SAMD1 modulates the function of KDM1A, possibly not just by influencing its recruitment to chromatin but also by influencing the catalytic activity of KDM1A." I wonder how this assay can discriminate whether the reduced me2 H3K4 levels are due to reduced histone/chromatin binding or reduced enzymatic activity?

As the histone proteins do not contain DNA, we assume that the reduced H3K4me2 levels are because SAMD1 is modulating the enzymatic activity of KDM1A. However, we agree with the reviewer, that our assay cannot fully distinguish between the effects on enzymatic or chromatin binding activity. Specifically, by binding to KDM1A, SAMD1 may change the conformation of KDM1A or its binding partners, and/or the composition of the KDM1A complex, which in turn may influence the chromatin association of the KDM1A complex. In our view, it is close to impossible to fully separate these two scenarios and would require much more sophisticated studies, such as Cryo-EM or Protein Crystallization to address this question. In the revised manuscript, we have modified the text to make this point clearer (lines 334-338).

Minor points:

5. Some of the analysis are not significant, this should be indicated in the text. Crucially, EMT signatures GSEA is not significant upon SAMD1 KO (Fig. 2c). Here maybe it helps to discriminate between up and down regulated genes, as e.g. Cdh1/Cdh2 would be counter regulated.

We thank the reviewer for the comment. We have added the information that the EMT pathway only trended (line 197). In addition, we now show the GSEA analyses of other mesenchymal-related gene sets, which are significantly affected (see Figure S5D). We also mention some more EMT-related genes that become upregulated in SAMD1 KO cells (lines 200-202). Further, we compared the gene expression changes upon SAMD1 KO from three different cell lines. Consistent with our initial hypothesis, we found that EMT pathway genes become only upregulated in the pancreatic cancer cell line, but not in liver cancer cells and mouse ES cells (see Figure S5G, H).

Regarding *CDH1*: *CDH1* is very lowly expressed and does not change upon SAMD1 deletion (based on the RNA-Seq data), suggesting that the absence of SAMD1 does not influence the balance between *CDH1* and *CDH2*. However, our results suggest that *CDH2* is the key downstream target of SAMD1 in these cells. The upregulation of *CHD2* appears to be sufficient to trigger the observed phenotype. So, despite the counter-regulation of *CDH1*/*CDH2* being commonly observed this appears to be not the case in our experimental setup.

That SAMD1 is influencing EMT pathways genes is also supported by mass-spectrometry analysis of extracellular vesicles that are secreted by wildtype and SAMD1 KO cells. Here, we also observe a strong upregulation of EMT-related proteins, including bone morphogenic proteins, Netrin and *WNT5A*. These results show a good correlation with the RNA-Seq data. We prefer to not include this data in the manuscript because we want to follow up on this aspect in future studies.

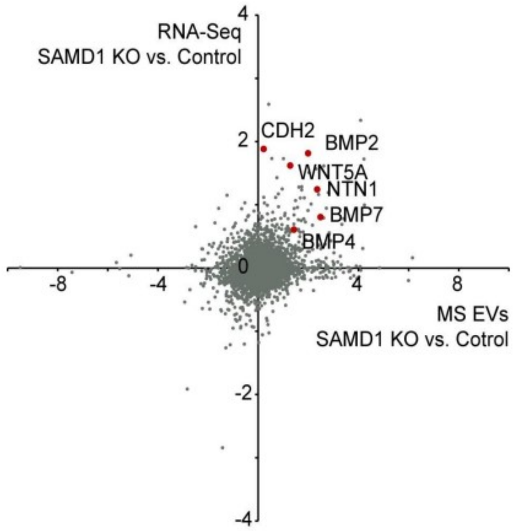

In addition, our initial analysis of the patient-derived data also supports a correlation between SAMD1 expression and EMT pathways (Figure 1B).

Overall, we are confident that SAMD1 KO leads to an increase of EMT-pathway genes in pancreatic cancer cells.

6. Are Fig. 2d,g and S4c the only result that can be extracted from the SAMD1 ChIP-Seq? It would be worth to correlate differential expression with SAMD1 peaks at CpG islands of the corresponding promoters.

We agree that more information can be extracted from the SAMD1 ChIP-Seq data. We now show the most enriched motif (comparing SAMD1 bound versus unbound CGIs) (Figure 2E), and we show that CpG islands bound by SAMD1 are in an active state, characterized by active histone mark and RNA polymerase II (Figure S6A). This result is consistent with the results we had obtained previously in mouse ES cells and HepG2 cells (PMIDs: 33980486; 35453756).
Regarding the correlation between differential gene expression and SAMD1 peaks: This is basically already shown by the GSEA analysis in Figure 2F (previous 2E) because it shows that the strongest SAMD1-bound genes become preferentially upregulated. However, we have now included an additional graph that compares the gene expression changes with promoter levels. This graph also shows that upregulated genes have typically higher levels of SAMD1 at the promoters. In addition, we show the SAMD1 profile on the respective gene promoters (see Figure S6C and D).

7. lines 258-261: The authors claim that "Consistently, via ChIP-qPCR, we observed an increase in H3K4me2 and H3K4me3 at the L3MBTL3 and CDH2 gene promoters upon SAMD1 deletion (Figure 3b)." However, the data indicate only an increase in me3 at the CHD2 promoter.

We agree with the reviewer and corrected this sentence in the text (lines 286-288).

8. How are the 250 SAMD1 target genes defined?

This point was mentioned in the Material and Methods under “Bioinformatic analysis”; “The top target genes were identified based on the SAMD1 ChIP-seq signal at promoters.” We have now added “250” to make clear that this sentence refers to the top 250 genes used in the GSEA analysis.

9. The supplemenetal figures need to be reorganized in the correct order: (2a-g, 3a-b), 4, 5, (3c-d, 2h-i)

We have now expanded and reorganized the supplemental figures. Now, everything should be in order.

Reviewer #2: I would like to congratulate the authors for the intense job. Here you will finde my comments hoping they improve your manuscript.

We highly appreciate the positive comment of the reviewer.

Minor revisions

About figure 1a. Around 20 months the difference among SMAD1 expression levels becomes relevant, but not before, regarding survival (if probability in Y exes means probability to be alive). Could the authors discuss this observation considering SMAD1 as a tumor progression marker?

The reviewer raises a good point. Indeed, the differences in the survival curves become more evident at later time points. We have now modified the text in the results (lines 109-112) and discussion (lines 522-526) to address this point and to mention that SAMD1 could potentially serve as a marker for tumor progression.

Major revisions

The quality of the figures is bad. I can't properly see the cells and descriptions. The quality of supplemental material is good.

We are not sure why the reviewer states that the figure quality is bad. Probably, the reviewer refers to the strongly compressed figures in the manuscript PDF (this compression is done by the journal and is outside the control of the authors). To access the high-resolution figures, it is possible to download them by clicking on “Click here to access/download” in the upright of the figures. We are confident that the quality of the figures is sufficient.

About Figure 1J. I am concerned about the rescue experiment using ER-SAMD1 fusion protein. How would you discard that migration properties do not belong to the ER part of the fusion protein?

We addressed these concerns by repeating the migration experiment with SAMD1 KO cells containing a FH-ER construct. Upon induction with 4-OHT, we could not detect any changes in the migration rates, concluding that the ER does not impact the migratory abilities of the cells. We included these findings in Supplementary Figure 4D, E.

How could the authors evaluate that the proteins determined in Figure 4 (b,c) are not bound to the ER part of the protein?

As indicated in Figure 4B, we used cells containing FH-ER only as a control. Thus we can exclude that the proteins detected in the SAMD1 IP are bound by the ER or the FH part of the protein.

Reviewer #3: In the manuscript by Liefke and colleagues, the authors examine the role of SAMD1 as a suppressor of tumor aggressiveness in PDAC at least in part via suppressing EMT as measured primarily through examining the levels of two EMT associated genes and examining migration. They also go on to show that SAMD1 is negatively regulated by FBXO11. The function of SAMD1 in PDAC appears to be different then that in other tumor contexts (where it is tumor promotional) and this finding, coupled with the FBXO11 regulation of SAMD1 provides novelty to the work. Overall, the experiments are rigorously performed and the data are well interpreted. Addressing the following issues would help to strengthen the paper:

We highly appreciate the positive and constructive comments of the reviewers. The responses to specific points are outlined below.

1. Since the major claim is that SAMD1 regulates EMT, a more comprehensive analysis of EMT is required. The authors should confirm a larger panel of EMT genes than just CDH2 and should also perform other studies of functionality associated with EMT (invasion, adhesion, polarity etc). The authors should also present the heat map of transcriptional changes in the EMT pathway with SAMD1 KO. Making a broad emt claim without more careful analysis is not warranted.

We now highlighted that other EMT genes except *CDH2* are dysregulated in SAMD1 deleted cells (Figure S5 D, E, text line 200-202). We have now included the heat maps of the EMT pathway, as well as two other mesenchymal pathways (Figure S5D). Note: these pathways are only upregulated upon SAMD1 KO in the PaTu8988t cells but not in HepG2 or mouse ES cells (Figure S5H)

The dysregulation of EMT-related factors is also supported by mass-spectrometry analysis of extracellular vesicles, which shows an upregulation of several important EMT proteins, such as *BMP2*, Netrin (*NTN1*) and *WNT5A* (see comment 5 of reviewer 1).

Moreover, we found a reduction of the tight junction protein 1 (*TJP1*, ZO-1) on mRNA, as well as on protein level (Figure S5F), demonstrating that the SAMD1 KO cells lose cell-cell contacts, which is another hallmark of EMT.

We also included adhesion and invasion assays, which clearly showed that the SAMD1 deleted cells adhere faster and have higher invasion rates compared to control cells (Supplementary Figure 2D-F).

We hope this additional work can convince the reviewer, that SAMD1 is involved in regulating the EMT pathway in PDAC cells.

2. The ER-fusion for SAMD1 does not appear to affect nuclear localization as much as binding to chromatin (based on the fractionation in suppl fig3B). Mechanistically- how is this working?

We now included a quantification of this figure (Figure S4C), which shows a strong difference in chromatin association with and without 4-OHT treatment. However, we cannot fully exclude some leakiness of the system, so a small amount of SAMD1 might be present

in the nucleus before induction. But importantly, we cannot detect any chromatin binding of SAMD1 without induction in ChIP experiments (see Figure 2M and Supplementary Figure 8B). For this reason, we are confident that this approach is suitable for our purposes.

3. Is it possible to show SAMD1 knock out is necessary for the EMT phenotypes by trying to induce an EMT with specific stimuli in the context of control vs KO cells? Can SAMD1 function in PDAC cells to prevent multiple forms of EMT induction?

To test this question, we treated the PaTu8988t cells with 10 ng/ml TNF to induce an EMT-like phenotype. The more common TGFbeta treatment for EMT induction was not possible in PaTu8988t cells, as the TGFbeta pathway is impaired due to a loss of SMAD4 (PMID: 37913894). We were able to induce some genes in both control and SAMD1 KO cells. Most of the genes were already upregulated upon SAMD1 KO and became stronger upregulated after TNF treatment compared to control cells. This suggests that the EMT phenotype can be triggered also in the presence of SAMD1, but the absence of SAMD1 makes the cells more susceptible to induction. In our view, this result indeed supports the hypothesis that the presence of SAMD1 can inhibit the induction of EMT phenotypes.
A more detailed study of this phenomenon would be of interest for further studies but is in our view beyond the scope of this manuscript.

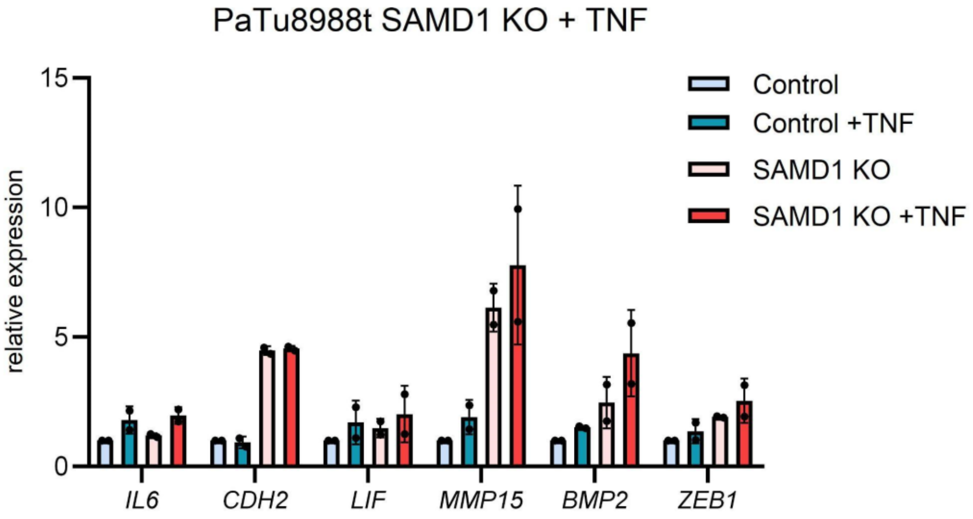

4. Why does increased binding to chromatin with FBXO11 deletion only impact specific genes (a small subset)?

If the reviewer refers to the chromatin binding SAMD1: As shown in the differential heatmap in Figure 6C about 80% of all SAMD1 target genes show an increase of SAMD1 (group 1 and 2) and only a smaller fraction shows no change (group 3) or a reduction (group 4). We show in Figure S10 that particularly genes with smaller CpG islands are affected by FBXO11 deletion. One could speculate that here the affinity of SAMD1 to the CpG island is weaker and therefore more susceptible to regulatory mechanisms, such as FBXO11.

If the reviewer refers to gene expression changes: The likely explanation is that an approximately 50% increase of SAMD1 chromatin binding is not sufficient to substantially change the gene expression of most of SAMD1 target genes. This is in contrast to the complete loss of SAMD1 upon SAMD1 KO, which can not be compensated. We have now addressed this point in more detail in the manuscript. (lines 482-486).

How does this relate to the EMT phenotypes (are the genes transcriptionally changed mostly EMT genes)?

We could not observe a strong change in the migration phenotype upon FBXO11 KO (see Supplementary Figure 10 G, H), possibly due to the minor effects on gene expression. We attempted to investigate this point in the opposite experiment, namely the overexpression of FBXO11. See point 5 for details.

Is there any identifying factor (sequence specificity- poteinally other binding partners) that would explain why only a small subset of genes in which binding changes are transcriptionally changed?

To address this question, one would need to investigate the gene expression changes upon FBXO11 KO at a global level. However, given that FBXO11 also plays many other roles, beyond SAMD1, it is likely that such an investigation will not provide a clear answer. Instead, it will likely be difficult to distinguish between effects that are related to SAMD1 and SAMD1-unrelated effects. FBXO11 plays a versatile role and is also involved in modulating gene expression by influencing the function of proteins such as BAHD1 (PMID: 33156908).

Also, we have found that although SAMD1 binds to thousands of genes, the effects on gene expression are relatively subtle, and only evident for a smaller number of genes. Even more challenging is the fact that the gene expression changes between distinct cell types are highly diverse, without hardly any common dysregulation genes. (The only exception is *L3MBTL3*, which we have found to be consistently upregulated upon SAMD1 deletion) (Supplementary Figure S5G). So far, we were not successful, in identifying an obvious connecting point between SAMD1 chromatin binding and its impact on gene expression. However, these phenomena are not uncommon for transcription factors and reveal the still major gaps in science in understanding how transcription factors influence the gene expression program.

5. How does FBXO11 affect EMT phenotypes (Migration etc)? This can be tested with the FBXO11 depleted cells.

We thank the reviewer for this important question. Investigation of the FBXO11 KO cells showed that the cellular migration is not affected (new Figure S10G, H). The likely explanation is that the 50% increase of SAMD1 chromatin binding in the FBXO11 KO cells is insufficient to substantially change the gene expression and, consequently, the cells' phenotypes. We have now discussed this point in more detail in the manuscript (lines 482-486).

In addition, we have performed the opposite experiment, namely the overexpression of *FBXO11*. Given that transient expression of FBXO11 in PaTu8988t cells leads to cell death, we performed the experiments in HEK293 cells, where we observed a reduction of SAMD1 chromatin levels at the investigated genes. This result is consistent with our working hypothesis that FBXO11 inhibits SAMD1 chromatin binding. However, again the 50% change in chromatin binding of SAMD1 does not appear to have a strong influence on the gene expression of individual genes (see Supplementary Figure 11 A, B).
As an alternative approach, we also established PaTu8988t cells that express an ER-FBXO11 fusion protein, similar to what we had done before for SAMD1. Upon induction of the nuclear localization of the ER-FBXO11 protein via tamoxifen, we observed a minor reduction of SAMD1 at some target genes. This effect is likely weaker compared to HEK293 cells because the expression of the FBXO11 protein is lower. Also, here we could not observe a change in gene expression. Due to this rather weak effect, we decided to not include these results in the manuscript.

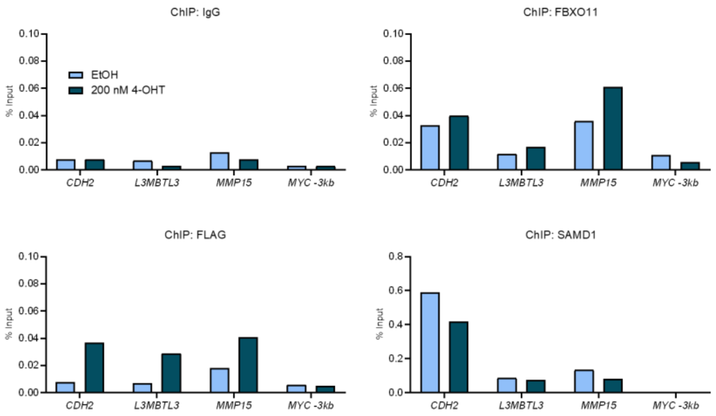

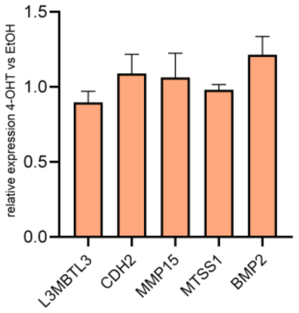

Notably, the idea that SAMD1 and FBXO11 have opposing functions on chromatin is supported by a recent comprehensive investigation of chromatin binding factors via mass spectrometry (PMID: 38448585). In the respective database (https://marcs.helmholtz-munich.de/readers/dna_methylation), SAMD1 is strongly repelled by methylated DNA (consistent with our original work), while FBXO11 surprisingly binds preferentially to methylated DNA (so far, no direct chromatin binding of FBXO11 has been described). One could speculate that FBXO11 is recruited to methylated DNA regions, where it influences SAMD1 and perhaps other chromatin-binding proteins.

Overall, there is significant novelty in working out this pathway but the paper remains incomplete in its assessment of how the SAMD1/FBXO11 axis is impinging on EMT to influence tumor aggressiveness.

We agree that the exact role of the SAMD1/FBXO11 axis in regulating EMT pathways and PDAC aggressiveness requires further investigations. In future work, we aim to address these points with additional experiments, including investigations of SAMD1's influence on the tumor microenvironment (TME) and via Pan02 orthotopic mouse models.

We hope the revised manuscript can convince the reviewer about the validity and importance of our work, and that it is appropriate for publication in PLOS Biology.
