# Supplementary material for: SAMD1 suppresses epithelial–mesenchymal transition pathways in pancreatic ductal adenocarcinoma
Source: PLoS Biol. 2024 Aug 13;22(8):e3002739. doi: 10.1371/journal.pbio.3002739 (PMC11343471; doi:10.1371/journal.pbio.3002739)
Supplement: Attachment — Submitted filename: Response to Reviewers.docx [file pbio.3002739.s020.docx]

We thank all three reviewers for evaluating our revised manuscript. Considering the comments of reviewer 3, we made following improvements:

1) We updated Figure 4B and marked all relevant proteins in MS experiment.
2) We added RT-qPCR experiments, where we measured EMT-related in two additional human PDAC cell lines, upon SAMD1 deletion. See Supplementary Figure S5F.
3) We compared the role of SAMD1 and FBXO11 in more cancer types, both regarding the correlation with EMT-pathway genes and patients’ survival. This is now presented in Supplementary Figure S12.
4) We added a limitation section to the end of the discussion to point out some weaknesses of our study.
5) We updated the S1 Data File, and S2 Table.

See below for a detailed response.

REVIEWS:

Reviewer #1: The authors addressed all my comments and improved the manuscript significantly. I like to congratulate the authors for their very nice work.

We thank the reviewer for the constructive comments on our original manuscript and are glad we could satisfy the reviewer with our revision.

Reviewer #2: Dear authors.

Thanks for considering all my request. Changes are sufficient for publication regarding my concerns. Good luck with the rest of the evaluation.

Best,

We thank the reviewer for the positive comments on our original manuscript and are glad we could satisfy the reviewer with our revision.

Reviewer #3: Overall, the authors have addressed most of my concerns from the first version of this manuscript. A few minor concerns remain:

We thank the reviewer for evaluating our revised manuscript, and we hope that our second revision can satisfy. Please find below the responses to the reviewer’s concerns.

1. The authors are arguing for context specific functions of SAMD1 in pancreatic cancer with respect to EMT, but only show effects on EMT gene signatures in one pancreatic cancer line (but not in other non-pancreatic cancer lines tested). To argue that SAMD1 has a specific anti-EMT function in pancreatic cancer, the authors should (at a minimum), show that they can identify some of the same EMT gene expression changes in other pancreatic cancer cell lines by qRT-PCR.

We fully agree with the reviewer’s point. We have therefore now performed RT-qPCR in two additional cell lines, BxPC3 and CFPAC, and measured several EMT-related genes upon SAMD1 KO. Consistent with the results from the PaTu8988t cells, we observed an increase of some of them in both cell lines. However, some of the genes are very lowly expressed in these cells and were excluded from the analysis.

In our view, these results support the notion that SAMD1 suppresses EMT-related genes in several independent PDAC cell lines.

2. Line 465 addresses enrichment of NEDD8, but I do not see this data in the figure.

We thank the reviewer for pointing this out. In fact, this information can be found in the supplementary Data file 1, where all underlying data are presented. However, to make this point clearer, we have now marked all relevant proteins in Figure 4B. This representation is likely better for the interested readership.

3. Line 554 says that increased binding of SAMD1 at the CDH2 promoter in the FBX011 KO cells- but this is not a significant change and thus the wording needs to be tempered.

We modified the text to make clearer that it is not significant. However, in all three replicates we observed an increase of SAMD1 chromatin binding, making us confident that FBXO11 deletion affects SAMD1 chromatin binding. We slightly modified the figure, to make this point clearer. That the result is not significant is only due to the variation between the experiments. (If one would normalize the data to the control cells, it would become significant).

4. While the authors do provide convincing data that FBX011 affects SAMD1 binding to chromatin, there is no clear functional consequence of this (at the gene expression or phenotypic level). This remains unfortunately one of the weaker parts of the manuscript- as it is unclear whether the effects of FBX011 on SAMD1 have any real consequences.

We agree that it is a bit unfortunate, that the effect of FBXO11, besides the effect on SAMD1 chromatin binding, appears to be relatively weak. However, we have now also analyzed the patient data of two more cancer types (Thymoma and cervical cancer), where low SAMD1 correlates with high EMT-pathway gene expression (Supplementary Figure 1). We find that also in these cancer types FBXO11 and SAMD1 expression oppositely correlate with EMT pathways. While low SAMD1 is linked to high EMT, the opposite is the case for FBXO11. Also, the patient survival data support an opposing role of these two proteins in these cancer types. We have now included this data as Supplementary Figure 12.

Thus, although we cannot conclude that FBXO11’s impact on SAMD1’s chromatin binding is of biological relevance, our biochemical data together with the patient data hint towards a functional interplay of these two proteins at least in some cancer types.

We have now added a limitation section to the manuscript to point out some weaknesses of our manuscript, including the point that our manuscripts lacks obvious evidence about the impact of FBXO11 towards the biological role of SAMD1. We hope these changes can satisfy the reviewer.

5. Line 651, I believe the authors meant "high FBX011" not "SAMD1"

We sincerely thank the reviewer for pointing out this mistake. It has been corrected.
