# Supplementary material for: SAMD1 suppresses epithelial–mesenchymal transition pathways in pancreatic ductal adenocarcinoma
Source: PLoS Biol. 2024 Aug 13;22(8):e3002739. doi: 10.1371/journal.pbio.3002739 (PMC11343471; doi:10.1371/journal.pbio.3002739)
Supplement: Attachment — Submitted filename: Response to Editorial Requests.docx [file pbio.3002739.s021.docx]

A) We would like to suggest a different title to improve accessibility. Please change your title to: “SAMD1 suppresses epithelial-mesenchymal transition pathways in pancreatic ductal adenocarcinoma”.

We have modified the title as wished.

B) Please make the deposited data publicly available. We have noticed that you have deposited your data, but that they currently are private. These need to be publicly available before the manuscript enters production.

We have made the data in GEO (GSE239418) publicly available. The mass-spectrometry data will be released, as soon as you provide us a DOI for the manuscript. (It is complicated to first release the data and subsequently add the DOI).

C) DATA POLICY:

You may be aware of the PLOS Data Policy, which requires that all data be made available without restriction: <http://journals.plos.org/plosbiology/s/data-availability>. For more information, please also see this editorial: <http://dx.doi.org/10.1371/journal.pbio.1001797>

Note that we do not require all raw data. Rather, we ask that all individual quantitative observations that underlie the data summarized in the figures and results of your paper be made available in one of the following forms:

1) Supplementary files (e.g., excel). Please ensure that all data files are uploaded as 'Supporting Information' and are invariably referred to (in the manuscript, figure legends, and the Description field when uploading your files) using the following format verbatim: S1 Data, S2 Data, etc. Multiple panels of a single or even several figures can be included as multiple sheets in one excel file that is saved using exactly the following convention: S1_Data.xlsx (using an underscore).

We provide all underlying data in the S1_Data.xlsx file, if applicable. See below for details.

2) Deposition in a publicly available repository. Please also provide the accession code or a reviewer link so that we may view your data before publication.

The ChIP-Seq and RNA-Seq are deposited to GEO (GSE239418) and have now been released.

The Mass spectrometry data will be released as soon as you provide us with a DOI for the manuscript. You can access the MS data beforehand with following account details:

Username: [reviewer_pxd044104@ebi.ac.uk](mailto:reviewer_pxd044104@ebi.ac.uk), Password: lb4cOc3O

Regardless of the method selected, please ensure that you provide the individual numerical values that underlie the summary data displayed in the following figure panels as they are essential for readers to assess your analysis and to reproduce it: Fig. 1A, Fig. S1A-C, Fig. S2E, Fig. S5C, Fig. S6A,D, Fig. S10A-C, EF and Fig. S12B

We have added the underlying data for

Fig. 1A, Fig. S1B, Fig. S1C, Fig. S12B (Kaplan Meier Plots)

Fig. 6B, Fig. S6D, Fig. S10E (ChIP-Seq Profiles)

Fig. S10A-C, Fig S10F, (Violin Plots)

For S1A, we provide the mean expression of SAMD1 in cancer versus normal tissue. The full dataset would include 9738 tumor and 8587 normal samples, which can be obtained from TCGA, if necessary.

We do not provide the underlying data for Fig S6A (ChIP-Seq heatmap) and Fig. S5C (GSEA), given that this would require to provide large datasets. The source of the ChIP-Seq data to create the heatmaps is provided in the Materials and Methods, and the underlying data for GSEA is available in the GEO repository.

The underlying data for Fig. S2E was already provided previously.

We added the new information about the supplementary data to “Supplementary Information” part. We also colored the sheets in the S1 Data file, for easier orientation.

NOTE: the numerical data provided should include all replicates AND the way in which the plotted mean and errors were derived (it should not present only the mean/average values).

For the numerical data, all replicates are provided in the S1 Data file. We added now “replicate x” to all data, where the replicate information was missing.

The way the data (mean and error) is presented, is described in the figure legends.

(We orientated ourselves on the supplementary data file from <https://doi.org/10.1371/journal.pbio.3002666>)

Please also ensure that figure legends in your manuscript include information on where the underlying data can be found, and ensure your supplemental data file/s has a legend. This includes references to repositories or papers for publicly available data.

We include the information where the underlying data can be found to each figure legend. We also added legends to the S1 Data file and information about repositories.

Please ensure that your Data Statement in the submission system accurately describes where your data can be found.

We confirm the correctness of the Data Statement.

D) CODE POLICY

Per journal policy, if you have generated any custom code during the course of this investigation, please make it available without restrictions. Please ensure that the code is sufficiently well documented and reusable, and that your Data Statement in the Editorial Manager submission system accurately describes where your code can be found.

Please note that we cannot accept sole deposition of code in GitHub, as this could be changed after publication. However, you can archive this version of your publicly available GitHub code to Zenodo. Once you do this, it will generate a DOI number, which you will need to provide in the Data Accessibility Statement (you are welcome to also provide the GitHub access information). See the process for doing this here: <https://docs.github.com/en/repositories/archiving-a-github-repository/referencing-and-citing-content>.

We did not generate any custom code.

As you address these items, please take this last chance to review your reference list to ensure that it is complete and correct. If you have cited papers that have been retracted, please include the rationale for doing so in the manuscript text, or remove these references and replace them with relevant current references. Any changes to the reference list should be mentioned in the cover letter that accompanies your revised manuscript.

The reference list is complete and correct.
